# Targeting Degradation of EGFR through the Allosteric Site Leads to Cancer Cell Detachment-Promoted Death

**DOI:** 10.3390/cancers11081094

**Published:** 2019-08-01

**Authors:** Melkon Iradyan, Nina Iradyan, Philippe Hulin, Artur Hambardzumyan, Aram Gyulkhandanyan, Rodolphe Alves de Sousa, Assia Hessani, Christos Roussakis, Guillaume Bollot, Cyril Bauvais, Vehary Sakanyan

**Affiliations:** 1 Scientific Technological Centre of Organic and Pharmaceutical Chemistry, Institute of Fine Organic Chemistry after A. L. Mndzhoyan, National Academy of Sciences of the Republic of Armenia, Yerevan 0014, Armenia; 2 Plate-forme MicroPICell SFR Santé F. Bonamy-FED 4203/Inserm UMS016/CNRS UMS3556, 44007 Nantes, France; 3 Scientific-Production Centre “Armbiotehnologiya”, National Academy of Sciences of the Republic of Armenia, Yerevan 0019, Armenia; 4 Départment de Biologie, Université Evry, INSERM, SABNP, Université Paris-Saclay, 91025 Evry, France; 5 Faculté des Sciences Fondamentales et Biomédicales, Université Paris Descartes, UMR 8601, CBMIT, 75006 Paris, France; 6 IICiMed, Faculté de Pharmacie, Université de Nantes, 44035 Nantes, France; 7 Synsight, 91000 Evry, France; 8 IICiMed, Faculté des Sciences et des Techniques, Université de Nantes, 44032 Nantes, France

**Keywords:** cancer, targeting chemotherapy, receptor tyrosine kinase, allosteric site, protein degraders, autophagy, cytoskeleton, anoikis, EGFR, Bim

## Abstract

Targeting epidermal growth factor receptor (EGFR) with tyrosine kinase inhibitors (TKI) has been widely exploited to disrupt aberrant phosphorylation flux in cancer. However, a bottleneck of potent TKIs is the acquisition of drug resistance mutations, secondary effects, and low ability to attenuate tumor progression. We have developed an alternative means of targeting EGFR that relies on protein degradation through two consecutive routes, ultimately leading to cancer cell detachment-related death. We describe furfuryl derivatives of 4-allyl-5-[2-(4-alkoxyphenyl)-quinolin-4-yl]-4H-1,2,4-triazole-3-thiol that bind to and weakly inhibit EGFR tyrosine phosphorylation and induce strong endocytic degradation of the receptor in cancer cells. The compound-promoted depletion of EGFR resulted in the sequestration of non-phosphorylated Bim, which no longer ensured the integrity of the cytoskeleton machinery, as shown by the detachment of cancer cells from the extracellular matrix (ECM). Of particular note, the longer CH_3_(CH_2_)n chains in the terminal moiety of the anti-EGFR molecules confer higher hydrophobicity in the allosteric site located in the immediate vicinity of the catalytic pocket. Small compounds accelerated and enhanced EGFR and associated proteins degradation during EGF and/or glutamine starvation of cultures, thereby demonstrating high potency in killing cancer cells by simultaneously modulating signaling and metabolic pathways. We propose a plausible mechanism of anti-cancer action by small degraders through the allosteric site of EGFR. Our data represent a rational and promising perspective in the treatment of aggressive tumors.

## 1. Introduction

Epidermal growth factor receptor, EGFR, belongs to a family of ErbB transmembrane proteins that govern signaling pathways and the expression of genes involved in the proliferation, survival, adhesion, and motility of cells [1]. A cognate ligand EGF binding to the extracellular region of EGFR results in receptor dimerization, leading to the activation of the ATP-binding site and autophosphorylation of tyrosine residues in the cytoplasmic region of the receptor. Mutations resulting in EGFR overexpression have been shown to be associated with the progression of different types of cancer; therefore, targeting this protein has been an important issue in medicinal chemistry in recent decades [2].

Potent ATP-competitive TKIs, including FDA-approved gefitinib, have been developed to inhibit the EGFR catalytic site [3]. TKIs with a covalent mode of action, such as canertinib, have also been designed to bind to a nucleophilic cysteine (Cys797) in proximity to the EGFR catalytic site [4]. Reversible and irreversible covalent inhibitors efficiently block the EGFR catalytic site and prolong the attenuation of downstream signaling cascades compared with competitive inhibitors [5]. However, a bottleneck of all potent EGFR TKIs is the acquisition of drug resistance mutations in the catalytic pocket or other sites of the receptor [6], as well as secondary effects, and low ability to attenuate tumor progression, as shown by numerous laboratory and clinical studies.

A clue to understanding the inhibition of the tyrosine kinase activity of EGFR by TKIs is the finding that the activation of the ATP-binding site is related to the action of hydrogen peroxide (H_2_O_2_) that is generated during cognate ligand binding to the receptor [7,8]. Indeed, the binding of EGF to EGFR promotes the transformation of O_2_ to H_2_O_2_ through the membrane-located NADPH oxidase Nox2; then, this reactive oxygen species reacts with Cys797, leading to the transition of the thiolate anion (Cys-S) to sulfenic acid (Cys-SOH), which is required for the activation of the ATP-binding site [8]. Recent data have shown that the amino acid Arg841 in the catalytic loop determines the dynamic structural alterations preceding the appropriate positioning of Cys797 to be sulfenylated [9]. Notably, H_2_O_2_ has also been shown to inhibit the catalytic site in protein tyrosine phosphatase PTP-1B; this inhibition contributes to enhancing the phosphorylation of the PTP-1B substrate EGFR [10].

Ligand-independent phosphorylation of EGFR has also been described in cells treated with small chemicals [11,12,13]. In particular, EGFR activation with 4-nitro-benzoxadiazole derivatives has been shown to rely on the generation of H_2_O_2_ by cytoplasmic superoxide dismutase in cancer cells [14]. Overall, these findings have suggested that the reactive hydrogen peroxide that is generated through a variety of metabolic reactions could unpredictably enhance the phosphorylation flux in EGFR-triggered pathways and could extinguish the effects of TKIs on cancer cells, thereby diminishing therapeutic efficacy. Therefore, anti-EGFR compounds that reduce receptor protein levels might be an alternative strategy to interrupt aberrant signaling in cancer cells.

EGFR endocytosis is an autophagic process, classified as microautophagy, which governs the fate of the receptor in cells [15]. Ligand-bound EGFR undergoes endocytosis followed by recycling and/or degradation of the receptor by proteolytic enzymes in lysosomes fused to endosomes [16]. Low doses of EGF activate clathrin-dependent endocytosis, whereas high doses of the cognate ligand promote both clathrin-dependent and clathrin-independent endocytosis [17]. Gefitinib has been shown to induce autophagy by suppression of EGF-stimulated endocytosis [18] and to induce atypical necroptosis in amino acid starved lung cancer cells [19].

EGFR phosphorylation status determines the functional state of integrins, which are transmembrane receptors that mediate cell adhesion to the ECM [20]. EGF binding to EGFR mediates the MAPK/ERK pathway leading to phosphorylation of the Bcl-2 interacting mediator (Bim), the sensor protein, which subsequently binds to microtubules and actin to provide the attachment of α/β-integrins to the ECM and the integrity of cytoskeleton [21]. Bim-mediated phosphorylation of the cytoskeleton is an integral part of anoikis, a programmed apoptotic death caused by cell detachment from the ECM [22]. The phosphorylation of EGFR-regulated signaling is dynamically affected by a variety of environmental factors, such as the availability of cognate ligands of the receptor or amino acids in a growth medium [23]. Other signaling and metabolic pathways also affect Bim phosphorylation at different sites of the protein isomers [24,25]. Compared with healthy cells, cancer cells possess a higher tolerance to anoikis, which is important for the metastatic progression of inflammatory tumors [26].

Therefore, there is a possible pharmacological window to disrupt aberrant signaling in tumors by targeting EGFR degradation with small chemical molecules. Recently, we synthesized polyfunctionalized heterocyclic compounds by combining three molecular scaffolds into one molecule that can bind EGFR [27]. Herein, we demonstrate that some compounds behave as weak inhibitors of EGFR phosphorylation and show enhanced degradation of the receptor and cytoskeletal proteins, leading to cancer cell detachment-related death. We provide a plausible explanation of the action of the compounds to highlight the rationale behind targeting EGFR degradation in anticancer therapy.

## 2. Results

### 2.1. Synthesis of Polyfunctionalized Heterocyclic Compounds

**Figure 1:**
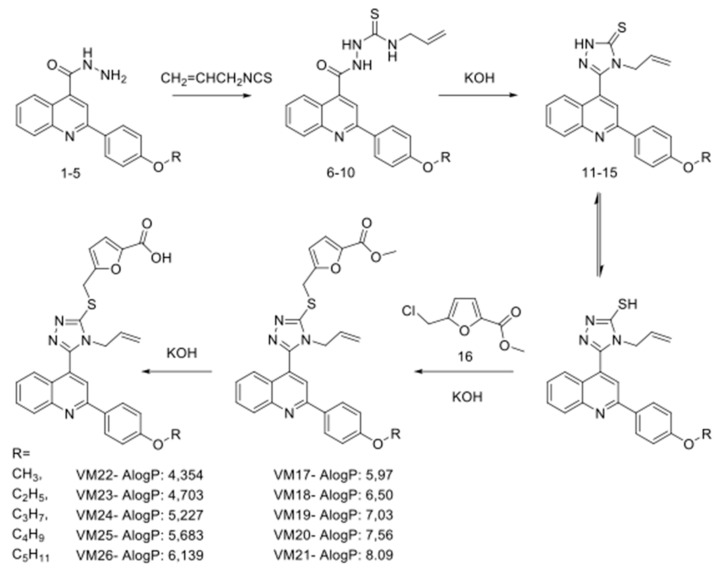
Synthetic route and structures of furfuryl derivatives of 4-allyl-5-[2-(4-alkoxyphenyl)-quinolin-4-yl]-4H-1,2,4-triazole-3-thiol. The AlogP values were calculated with Discovery Studio 4.0 software.

Furfuryl derivatives of 4-allyl-5-[2-(4′-alkoxyphenyl)quinolin-4-yl]-4H-1,2,4-triazol-3-thiol were synthesized by combining quinoline, furan and triazole scaffolds in a single molecule, suggesting that the action of the novel compounds will differ from known anti-EGFR TKIs [27]. To intensify this difference, we connected alkyl ether substituents of different lengths to the benzene ring at the extremity of new structures, which might enhance local hydrophobicity of the bound EGFR and sensitize the receptor protein to the action of proteases. The synthetic route of compounds is shown in Figure 1. 

The starting materials for synthesis were hydrazides of 2-(4-alkoxyphenyl)quinolin-4-carboxylic acids (1–5) [28], which were delivered in conjunction with allyl isothiocyanate in ethanol, resulting in the synthesis of N4-allylthiosemicarbazide 2-(4-alkoxyphenyl)quinolin-4-carboxylic acids (6–10). These compounds were further cyclized with KOH into 4-allyl-3-[2-(4-alkoxyphenyl)quinoline-4-yl]-4,5-dihydro-1H-1,2,4-triazole-5-thiones (11–15) [29]. The reaction of compounds 11–15 with the methyl ester of 5-chloromethylfuran-2-carboxylic acid in the presence of equimolar amounts of KOH yielded only S-substituted derivatives (VM17-VM21). In the latter structures, the ester group was hydrolyzed with potassium hydroxide in water-methanol medium to a carboxyl group (VM22-VM26).

### 2.2. Identification of Compounds That Decrease EGFR Levels in Cancer Cells

**Figure 2:**
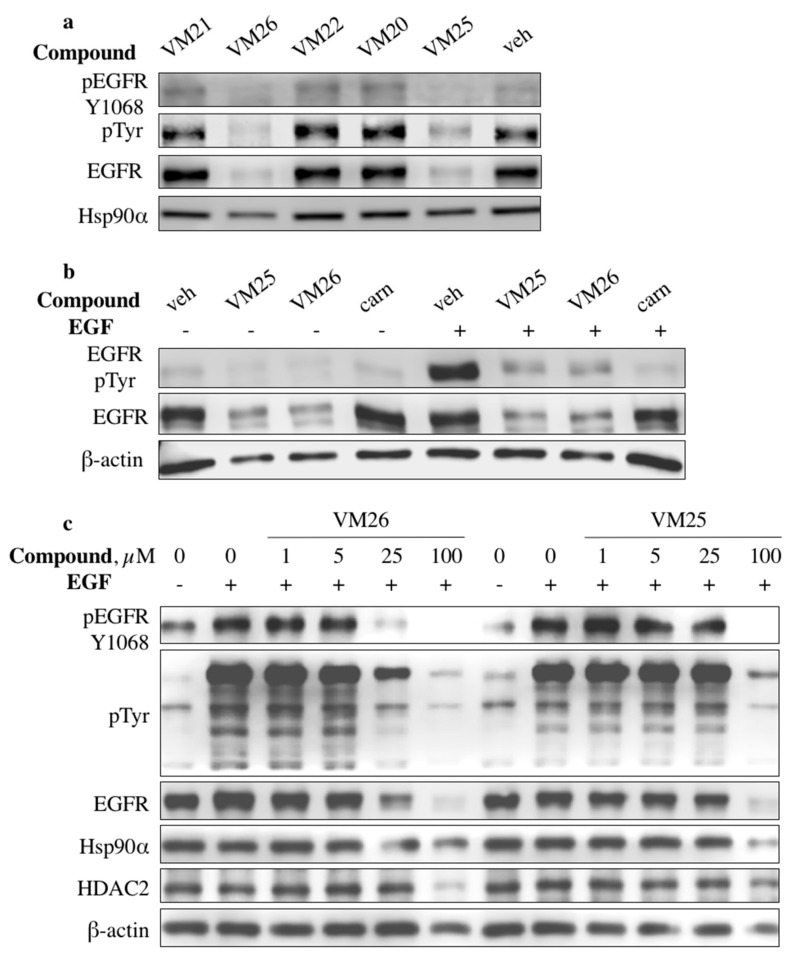
Small compounds decrease epidermal growth factor receptor (EGFR) expression in MDA-MB-468 cells. (**a**) Serum-starved MDA-MB-468 cells were incubated with vehicle (veh) and selected compounds at a concentration of 100 µM for 2 h and then stimulated with 200 ng/mL EGF for 10 min. (**b**) Comparison of the action of the compounds VM25 and VM26 with canertinib (1 µM). (**c**) Compounds VM25 and VM26 exhibit dose-dependent effects on EGFR expression.

To assess the ability of new compounds to modulate EGFR expression and phosphorylation, we used the triple-negative breast cancer cell line MDA-MB-468, in which the receptor protein is overexpressed compared with the low expression of its counterpart ErbB2 [30]. The cells were grown in DMEM deprived of fetal bovine serum (FBS) overnight and treated with 100 µM of the compounds followed by EGFR stimulation with its cognate ligand EGF. Western blotting revealed that two compounds, VM25 and VM26, significantly suppressed tyrosine phosphorylation and decreased receptor expression levels (Figure 2a). No effects were detected with compounds VM20 and VM21, which contain the ester group instead of the carboxylic acid in VM25 and VM26, respectively (Figure 1). In cells incubated with compound VM25 or VM26 without further stimulation with EGF, EGFR levels decreased compared with the cells incubated with vehicle (0.1% dimethylsulfoxyde, DMSO) (Figure 2b). A covalent inhibitor of EGFR, canertinib, which was used as a control, suppressed tyrosine phosphorylation of EGFR without reducing EGFR levels under the conditions used.

A comparative evaluation of the two active compounds showed that the phosphorylation levels at Tyr1068 and EGFR expression decreased in a dose-dependent manner in serum-starved MDA-MB-468 cells treated with the compounds and then stimulated with EGF (Figure 2c). The cells also gradually suppressed the tyrosine phosphorylation of other protein kinases, as detected with the anti-pTyr antibody, and compound VM26 was more active than VM25. Compound VM26 was also active in non-small-cell lung-cancer (NSCLC) A549 cells (Supplementary Figure S1). Notably, the decrease in EGFR levels was accompanied by a decrease in the levels of other proteins, such as Hsp90α, HDAC2, and β-actin, in cells treated with higher concentrations of the compounds (Figure 2c and Supplementary Figure S1).

The ability of the active compounds VM25 and VM26 to simultaneously suppress tyrosine phosphorylation and to reduce EGFR levels and other functionally unrelated proteins suggested that the small molecules induce protein degradation by first targeting EGFR in cancer cells.

### 2.3. Small Compounds Activate Endocytosis of EGFR

**Figure 3:**
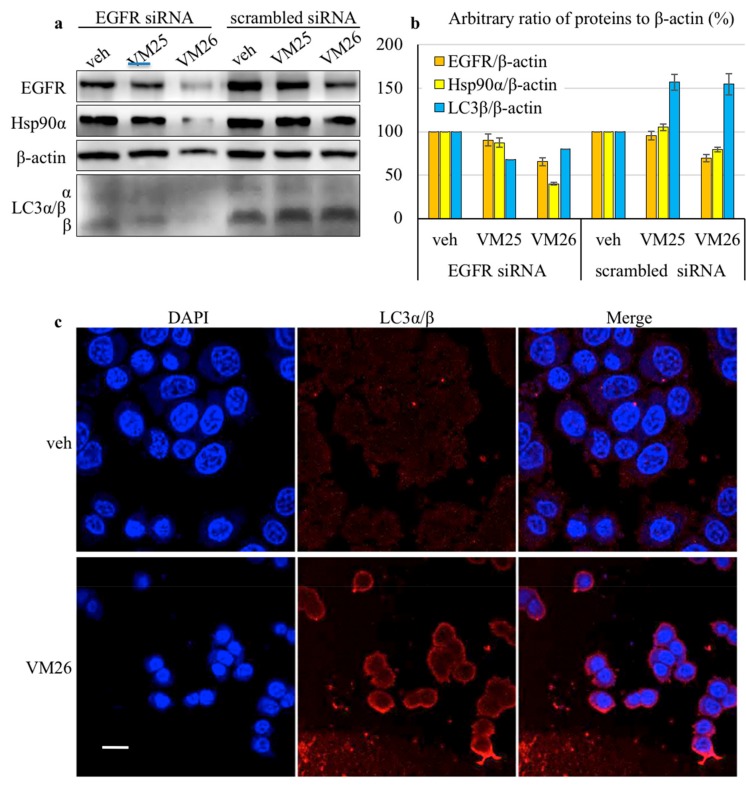
Small compounds activate microautophagy in cancer cells. (**a**) Western blot of proteins extracted from MDA-MB-468 cells after transfection with EGFR siRNAs or scrambled siRNAs and exposure to 25 µM VM25 and VM26 for 2 h in serum-deprived Dulbecco’s Modified Eagle Medium (DMEM). (**b**) Relative levels of proteins were estimated as the ratio to β-actin and adjusted to the vehicle-treated cells normalized to 100%. (**c**) Immunofluorescence detection of LC3α/β in MDA-MB-468 cells exposed to 2.5 µM VM26 for 1 h. The size bar is equal to 10 μm.

MDA-MB-468 cells were transfected with EGFR-specific siRNA and used to assess the protein levels after exposure to the small compounds. Despite the moderate efficacy of siRNA silencing (apparently related to the high copy number of EGFR in the MDA-MB-468 cell line), the cells displayed a remarkable decrease in EGFR and Hsp90α levels and less in β-actin levels when exposed to compound VM26 (this decrease was less apparent with compound VM25) in serum-deprived culture compared with the vehicle (Figure 3a). In this assay, we also assessed the ubiquitin-like proteins LC3α/β by taking into consideration that LC3β accumulation correlates with increasing numbers of autophagosomes and therefore, it can be used for monitoring autophagy [31]. A relatively intense 16-kDa band corresponding to LC3β was detected in the cells, indicating the activation of EGFR endocytosis. The shift of LC3α to LC3β was more pronounced with scrambled siRNA than with EGFR-specific siRNA in cells treated with small compounds, suggesting that knockdown of the receptor reduced the autophagy response. The quantification of the ratio of LC3β to β-actin showed that the relative levels of the autophagy biomarker were higher by approximately 50% after exposure to the compounds VM25 or VM26 in cells transfected with scrambled siRNA compared with the vehicle (Figure 3b). Hence, compounds VM25 or VM26 could induce the degradation of EGFR and associated nutrient-prone proteins in cancer cells during growth in the serum-deprived medium.

To verify that the activation of LC3β was a result of the action of small compounds, the FBS-starved cells were incubated with a lower concentration of VM26 for a shorter time, and the autophagy biomarker was analyzed with immunofluorescence microscopy. The LC3β protein emitted a strong fluorescent signal upon exposure to VM26 relative to the vehicle (Figure 3c), indicating that the penetration of a small compound resulted in a rapid response of the autophagy mechanism in cells. Indeed, the spots, clearly visible near the plasma membrane on the enlarged image (Supplementary Figure S2), represent likely autophagosomes that could be formed by invagination of small compound/EGFR-bound complexes at the early stages of microautophagy of the transmembrane receptor.

**Figure 4:**
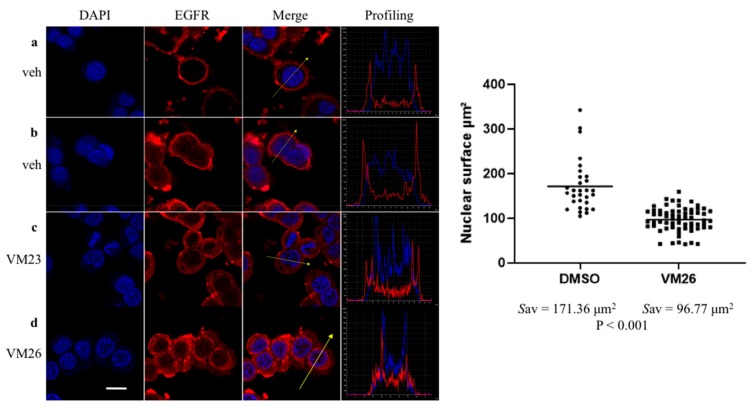
EGFR rapidly responds to the chemical invasion of small compounds in cancer cells. Immunofluorescence detection was carried out with anti-EGFR mAb (the epitope in the extracellular domain). Fluorescence in nonpermeabilized (**a**) and permeabilized (**b**) cells after exposure to vehicle and in permeabilized cells after exposure to VM23 (**c**) and VM26 (**d**). MDA-MB-468 cells were incubated with VM23 and VM26 as described in Figure 3c. Profiling curves of fluorescent signal distribution in representative cells are shown with dashes in merge. (**e**) Boxplot histogram of a 2D square of nuclei surfaces upon exposure to vehicle and VM26. *S*av is the average square of the nuclear surface in cells. The size bar is equal to 10 μm.

Next, immunofluorescence imaging confirmed that small compounds specifically target EGFR in starved cancer cells. In control experiments with the vehicle, the receptor was found exclusively in the cytoplasmic membrane of nonpermeabilized cells or predominantly in the cytoplasmic membrane of permeabilized cells (Figure 4a,b and Supplementary Figure S3a,b). In contrast, after a short exposure to low concentrations of VM23 or VM26, the fluorescence signal emitted by EGFR decreased in the cytoplasmic membrane and increased in the cytoplasm (Figure 4c,d and Supplementary Figure S3c,d). Moreover, the second fluorescent area illuminated around nuclei was identified as two new peaks on signal profiling curves (to the right in the images). The dispersion of fluorescence signals in the cytoplasm, as well as alternating low peaks on profiling curves, indicated that a portion of EGFR is released in this compartment. Notably, these features were associated with the reduction of the size of cells and nuclei upon exposure to the compounds. The measurement of the two-dimensional axis of nuclei showed a statistically significant decrease in their size and surface square after exposure to VM26 compared with the vehicle (Figure 4e and Supplementary Table S1). This morphological change strongly suggested that exposure to small compounds rapidly results in the diminution of the cell contacting surface with the plate surface, which in the biological sense indicates the loss of the adhesion capacity of cells to the ECM.

### 2.4. Small Compounds Promote Cancer Cell Detachment

We noticed that incubation of cancer cells with high concentrations of compounds in FBS-deprived medium for approximately two hours lowered the levels of β-actin, which was used as a loading control in Western blot experiments (Figure 2). Given that β-actin is a key cytoskeletal protein, we assumed that the analysis of cell extracts with the same total protein concentration to be loaded does not reflect the actual protein levels in a typically adherent cell line, such as MDA-MB-468, if cells detach from the plate surface, mimicking ECM. Therefore, we decided to load the same volumes of cell extracts, regardless of the total protein concentration, in order to better elucidate the subsequent processes caused by the depletion of EGFR in the attached cells and detached cells after treatment with chemical compounds. 

The MDA-MB-468 cells were grown in serum-deprived DMEM overnight and then treated with compounds VM23, VM25, VM26, and gefitinib for 6 h. The culture medium covering the cells attached to the well surface was carefully transferred to tubes; the wells were gently washed with warm PBS, and the wash solutions were collected in the same tubes. The collected samples were centrifuged, and the pellets containing the putatively detached cells were treated with lysis buffer. Cells attached to the surface of wells were separately collected and lysed. To increase the detection sensitivity of proteins in putative detached cells, lysis was performed in smaller volumes compared with the attached cells.

**Figure 5:**
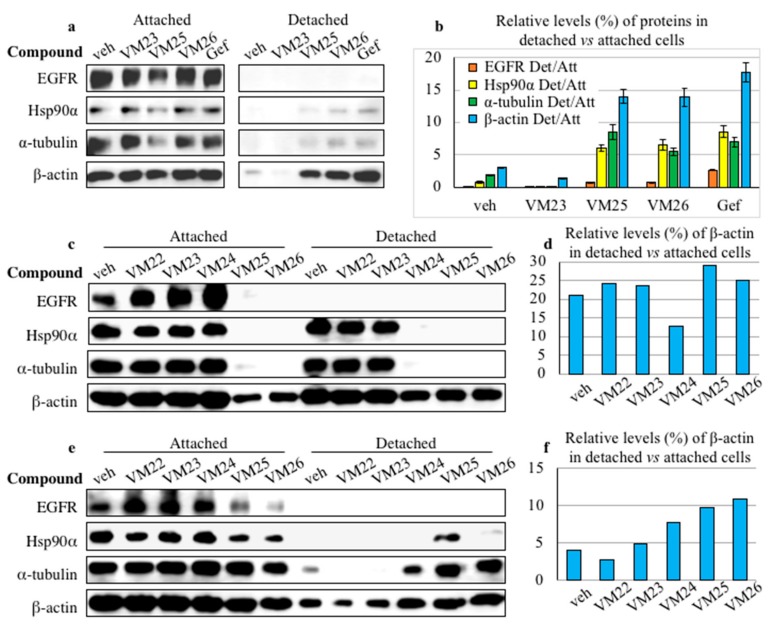
Small compounds promote detachment of cancer cells. MDA-MB-468 cells were treated with 25 µM of the compounds VM23, VM25, VM26 or gefitinib (Gef) in serum-deprived medium for 6 h (**a**), with 25 µM of the compounds VM22-VM25 in serum-deprived medium for 48 h (**c**) or with 50 µM of the compounds VM22-VM25 in FBS-supplemented medium for 48 h (**e**). The relative levels (%) of proteins in detached cells were estimated as a ratio of the detached cells (Det) to the attached cells (Att) (**b**) or β-actin (**d**,**f**). The detached cells were lysed in one-quarter (**a**,**b**) or one-tenth of the volume of the attached cells (**e**). The differences in the volume of lysed buffer used were considered to normalize the abundance of proteins.

The cytoskeleton proteins β-actin and α-tubulin, Hsp90α, and traces of EGFR were detected in the culture medium collected from the attached cells, thereby proving the detachment of the cells treated with compounds VM25 and VM26 or gefitinib for comparison (Figure 5a). As the loading control of total proteins was not an appropriate means to assess the cell detachment, we used a ratio of each protein in potentially detached cells to the attached cells treated with a given compound for evaluation. Quantitative estimation of relative levels of individual proteins in the same cultures revealed higher levels of the proteins in the detached cells after exposure to VM25, VM26 and gefitinib compared with VM23 or vehicle (Figure 5b). Curiously, the detachment of cancer cells promoted by the potent TKI gefitinib (Figure 5a) was not described in a large number of studies when the amount of total protein in cell extracts was adjusted to the same concentration to be loaded.

To compare the ability of five compounds, VM22-VM26, to detach the cells, the treatment in serum-deprived medium was prolonged for 48 h. The cells treated with VM22, VM23 or vehicle displayed similar profiles of EGFR, Hsp90α, α-tubulin and β-actin in the attached cells, and except EGFR, the three other proteins were detected in the detached cells (Figure 5c). In contrast, the cells treated with VM25 or VM26 displayed almost no EGFR, Hsp90α, and α-tubulin and significant decrease in β-actin levels in the attached cells. Moreover, none of the first three proteins was detected in the detached cells as an indication of cancer cell death.

The ability of the compounds to promote the detachment of cells was also studied after 48 h of growth in DMEM supplemented with FBS. Given that FBS contains a broad spectrum of growth factors, including EGF [32], the cells were incubated with higher concentrations of the compounds VM22-VM26. No significant differences were detected in the protein levels in the attached cells treated with VM22, VM23 or vehicle (Figure 5e). In contrast, compounds VM25 and VM26 exhibited an elevated capacity to decrease the expression levels of EGFR, Hsp90α, and α-tubulin in attached cells.

In attempts to quantitatively assess the efficiency of cell detachment after longer exposure to the compounds, we estimated the ratio of β-actin in the detached cells versus the attached cells, as only this protein was detected in all 48-h treated cell samples. In the medium supplied with FBS, cell detachment was clearly detected with VM24, VM25, and VM26 (Figure 5f), whereas the effect of these compounds appeared to be less evident after growth in serum-deprived medium (Figure 5d). Obviously, the abundance of only β-actin in the detached cells underestimates the efficiency of the compounds, as higher stability of β-actin or re-adhesion of survived cells could misrepresent the actual situation.

In fact, small compounds exhibited moderate to low toxicity in MDA-MB-468 cells (Supplementary Table S2). The toxicity of the active compounds VM25 and VM26 was similar to that of gefitinib and close to values determined previously for this potent TKI by other authors [33]. The kinetics of the cell viability in complete media indicated a time-dependent killing effect of the compounds VM25 and especially VM26 on MDA-MB-468 and A549 cultures during a 72-h incubation (Supplementary Figure S4).

Taken together, these results showed that endocytic degradation of EGFR leads to depletion of the receptor, which drastically destabilizes the cytoskeleton, leading to the detachment of cancer cells from the ECM and, ultimately, to death, resembling apoptotic anoikis.

### 2.5. Bim Expression is Temporarily Up-Regulated in Starved Cancer Cells upon Exposure to VM26

**Figure 6:**
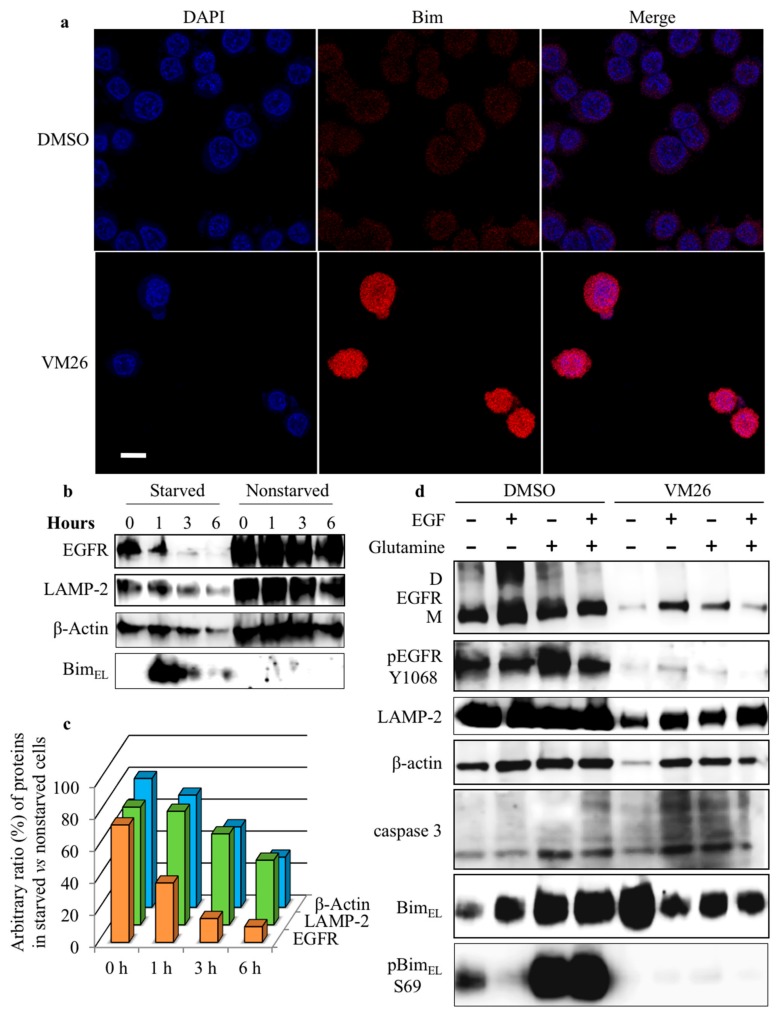
The compound VM26 promotes sequestration of Bim in MDA-MB-468 cells. (**a**) Bim response to the compound VM26. Treatment conditions were similar to Figure 3c; the immunofluorescent detection was carried out in permeabilized cells with anti-Bim mAb. (**b**) Kinetics of protein expression in the cells treated with 25 µM VM26. (**c**) Relative levels of proteins were estimated as their ratio in starved cells to nonstarved cells considering 100% of each protein in nonstarved cells. (**d**) Impact of the addition of EGF (200 ng/mL) and glutamine (2.0 mM) on protein profiling in cells left untreated or treated simultaneously with 25 µM VM26 for 6 h in FBS-deprived medium. Proteins were extracted in moderate-strength lysis buffer. The EGFR monomer (M) and dimer (D) are shown.

To determine whether the destabilization of cytoskeleton machinery by the compounds is related to the status of the sensor protein Bim, protein expression was assessed first with immunofluorescence imaging. The fluorescent signal emitted by Bim was significantly enhanced in the cells at exposure to 2.5 µM VM26 for one hour compared with the vehicle (Figure 6a).

This observation was verified by studying the kinetics of Bim expression and phosphorylation in 12-h FBS-deprived cultures incubated further with 25 µM VM26 for 6 h in FBS-deprived and nondeprived media. The MDA-MB-468 cells treated in FBS-deprived medium displayed essentially increased Bim_EL_ expression after one-hour exposure to the compound, but the protein level decreased after a longer, three-hour exposure (Figure 6b). Notably, high levels of Bim_EL_ were associated with an essential decrease in the abundance of EGFR after one-hour exposure to VM26. The expression of the lysosomal protease LAMP-2 and the cytoskeletal protein β-actin declined later compared with EGFR in the starved culture. In parallel series of experiments with nonstarved MDA-MB-468 cultures, the Bim_EL_ level was low and the decrease in the levels of other proteins could be observed after a longer, six-hour incubation with VM26.

The efficacy of VM26 on the degradation of EGFR, LAMP-2, and β-actin was quantified by comparing a ratio of corresponding proteins in starved cells versus nonstarved cells (Figure 6c). The histograms clearly demonstrate that the reduction in the ratio of EGFR in the starved cells is faster and substantially greater compared with β-actin. This two-speed decrease in the abundance of proteins obviously reflected two processes, the early and rapid endocytic degradation of EGFR followed by a slower disintegration of the cytoskeleton due to Bim sequestration.

### 2.6. Glutamine Starvation Enhances Small Compound-Promoted Sequestration of Bim

To clarify the underlying factors involved in small compound-induced sequestration of Bim, protein profiles were assessed with only compound VM26 and compared after the addition of its mixture with EGF or glutamine or both into a 24-h the FBS-deprived medium containing overnight starved cultures and cultivation continued for 6 h.

The analysis of the protein abundance in control assays with the vehicle showed that six-hour cultures of MDA-MB-468 display slightly higher expression levels of β-actin after the addition of EGF, glutamine or both, and the increase levels of EGFR monomer and dimer forms after the addition of EGF (Figure 6d). Meanwhile, glutamine and the mixture of EGF with glutamine, and to a lesser extent EGF, increased the expression of Bim_EL_ compared with the vehicle or EGF alone. Moreover, a significant increase in the rate of phosphorylation was detected at Ser69 of Bim_EL_, which is associated with the enhanced expression of the sensor protein, and likely with the enhanced phosphorylation at Tyr1068 of EGFR. Therefore, the completion of the medium with a fresh portion of glutamine improves the functional state of Bim_EL_.

The cells treated with VM26 in the presence of EGF, glutamine or both displayed an increase in the expression levels of EGFR, LAMP-2, β-actin, and a cleaved caspase 3 compared with low expression of the proteins in the presence of the compound (Figure 6d). The expression of Bim_EL_ essentially increased after exposure of cells to VM26, and no reasonable modulation was detected after addition of EGF, glutamine or the mixture EGF and glutamine to the medium. However, in contrast to the results of control assays with vehicle, VM26 strongly suppressed Ser69 phosphorylation in Bim_EL_, likely associated with the decreased phosphorylation in EGFR, regardless of the addition of EGF, glutamine or both to the growth medium.

We also assessed the effect of VM26 on the DU-145 prostate cancer cell line, characterized by an unusual decrease in EGFR level in response to EGF action in the autocrine loop, presumably associated with endocytosis [34]. Expression levels of EGFR decreased one hour after the addition of EGF or VM26 in serum-deprived RPMI-1640 (Supplementary Figure S5a). In both cases, depletion of the receptor did not affect LAMP-2 and β-actin levels, whereas it increased Bim_L_ and Bim_EL_ levels and stimulated Ser69 phosphorylation. However, the depletion of EGFR under the action of VM26 for three hours resulted in a decrease in Bim_EL_ expression and possibly Ser69 phosphorylation in the attached cells. In the meantime, a greater number of cancer cells appear to detach from the ECM, compared to the vehicle (Supplementary Figure S5a,b).

The VM26-promoted detachment of cancer cells, associated with depletion of EGFR, was particularly apparent with prolonged treatment. After a 24-h incubation, VM26 resulted in the disappearance of EGFR and β-actin, and only traces of LAMP-2 or Bim could be detected in the attached cells, regardless of the addition of EGF or glutamine in the starvation medium (Supplementary Figure S5c).

These results highlight the efficacy of VM26 in the dramatic degradation of EGFR in broad levels of receptor gene expression in MDA-MB-468 and DU-145 cancer cells during EGF starvation. It can be assumed that the medium devoid of FBS becomes also deprived of glutamine with longer growth of cancer cells. The dual starvation for EGF and glutamine makes cells more vulnerable to VM26 invasion, thereby accelerating compound-induced EGFR depletion and increasing cytoskeleton instability caused by Bim sequestration.

### 2.7. Compound VM26 Weakly Inhibits EGFR Phosphorylation

**Figure 7:**
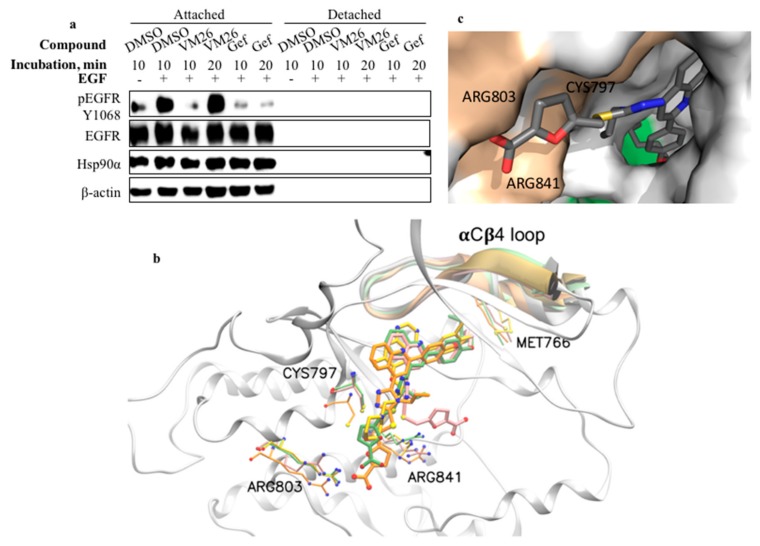
Compound VM26 weakly suppresses EGFR phosphorylation. (**a**) EGFR expression and tyrosine phosphorylation at Tyr1068 were evaluated in MDA-MB-468 cells after exposure to 25 µM VM26 or gefitinib (Gef) for 10 min and 20 min. The cells were stimulated with EGF (200 ng/mL) for 5 min. Modeling of interactions between representative small compounds and the EGFR catalytic domain. (**b**) A 3D view of the superposition of EGFR bound to VM23 (orange), VM25 (pink), and VM26 (green) and gefitinib (yellow). Nitrogen, oxygen, and sulfur atoms are represented as blue, red and yellow spheres, respectively. The αCβ4 loop containing Met766 corresponds to the region between 761 and 781 amino acid positions. (**c**) Close-up view of the compound VM26 accommodation in the hydrophobic cavity (green) in the tyrosine kinase domain. The amino acids Met766, Phe856, Leu788 and Leu858 form the cavity, and Cys797, Arg803, and Arg841 are located in the catalytic pocket (salmond) (see also Supplementary Figure S4).

We examined whether the suppression of EGFR phosphorylation resulted from only the protein degradation or whether the small compound also inhibited the tyrosine kinase site. MDA-MB-468 cells grown in serum-deprived medium were incubated with the most active compound, VM26, for short time periods, 10 and 20 min, and then, EGFR phosphorylation was stimulated by the incubation of the cells with EGF for 5 min. The decrease in tyrosine phosphorylation at Tyr1068 was detected only after 10-min exposure to VM26, whereas gefitinib, used as reference TKI, strongly inhibited EGFR phosphorylation at both 10-min and 20-min exposure times (Figure 7a). Therefore, the ability of VM26 to transiently prevent tyrosine phosphorylation of EGFR by the cognate ligand EGF characterizes the compound as a reversible and weak ATP competitor that shows inhibitory potency only within a short time.

### 2.8. Binding of Small Compounds Results in Dynamic Structural Rearrangements in EGFR

The compounds VM22-VM26 perfectly docked in the tyrosine kinase catalytic pocket of EGFR of the highly resolved 3D structure 3W32 [35] by demonstrating negative free energy values comparable to gefitinib (Supplementary Table S3). The analysis of the VM23, VM25, and VM26 interactions showed a similar set of virtual contacts with amino acids in the best-docked conformations (Supplementary Table S4).

To examine the binding process of similar molecules to EGFR, we employed molecular dynamics (MD) simulations. The root mean square deviation (RMSD) of the molecular interactions was monitored between EGFR and the compounds VM23, VM25, and VM26 and the well-characterized drug gefitinib for comparison. The RMSD curves of the free molecules and the EGFR protein backbone were stable throughout monitoring for 500 ns of simulation (Supplementary Figure S6). The virtual VM23/EGFR complex was stable until the end of the simulation. Conversely, a remarkable shift was monitored in the dynamics of VM26 binding to the protein at 100,000 ps and VM25 binding at 300,000 ps, indicating structural rearrangements in the compound-protein complexes. No shifts were detected with VM23 and gefitinib under the conditions used despite of rather similar distances between the molecules and the selected amino acids in the bound structures (Supplementary Table S5).

At the beginning of the interactions, the carboxylate group in the furan moiety of VM23, VM25 and VM26, which is exposed to solvent, establishes tight contacts with the guanidinium moiety of Arg803 located in the catalytic pocket (Figure 7b and Supplementary Figure S7). The furan moiety of VM23 also makes contacts with Cys797 and Arg841; this configuration of the bound protein is not subject to structural changes, at least within 500 ns. In contrast, the carboxylate in the extremity of the furan moiety in VM26 turns from Arg803 to Arg841, leading to the formation of a hydrogen bond. Moreover, the furan and imidazole moieties in VM26 establish interactions with Cys797. Similarly, in compound VM25, interactions between the imidazole moiety and Cys797 are associated with delayed dynamic rearrangements, which shift the binding from Arg803 to Arg841. The alkyl chain at the extremity of the three molecules is accommodated within a hydrophobic pocket terminated by Phe856 (Figure 7c, Supplementary Figure S7 and Supplementary Video S1). The short CH_3_CH_2_ chain of VM23 is not bulky enough to fill the hydrophobic pocket, whereas the longer CH_3_(CH_2_)_4_ chain of VM26 almost completely occupies the pocket. 

Crucially, the position of this hydrophobic pocket corresponds to the allosteric site discovered recently in EGFR-bound complexes with two molecules, EAI001 and EAI045 [36]. Our docking analysis revealed that the compound VM26 shares similar amino acid interactions with EAI045 bound to the wild-type EGFR (PDB ID 2GS7) and the mutant T790M/V948R (PDB ID 2JIV), according to the best-score generated conformations (Supplementary Figure S8, Supplementary Table S6). As far as VM22–VM26 compounds are larger, they establish more amino acid contacts, which could increase the binding affinity for EGFR (Supplementary Table S7). 

## 3. Discussion

This work demonstrates an alternative strategy to interrupt the aberrant EGFR-mediated phosphorylation cascade in cancer through authentic degradation of the receptor rather than strong inhibition of the tyrosine kinase site. The furfuryl derivatives of 4-allyl-5-[2-(4-alkoxyphenyl)quinolin-4-yl]-4H-1,2,4-triazole-3-thiol reversibly and weakly bind to the catalytic site of EGFR and induce protein degradation, resulting in the detachment of cells from the ECM. Given that the binding of our molecules to the catalytic pocket extends into the allosteric site, they can be categorized as anti-EGFR molecules of the fourth generation, to which belong recently described TKIs, EAI001 and EAI045 [37]. The druggable potency of compounds VM22-VM26 relies on two distinct routes, started by EGFR endocytosis and continued by Bim sequestration-mediated disintegration of the cytoskeleton. Obviously, the compound-induced endocytosis of EGFR is of particular importance, as the depletion of significant amounts of the receptor has detrimental consequences for cell viability. 

The cytoskeleton machinery is composed of polymers of actin and microtubules formed by tubulin, which in concert with other proteins allow integrins to attach cells to the ECM [38]. Bim is a sensor protein that initiates the intrinsic apoptotic pathway in healthy and diseased cells, and its phosphorylation status, which is required for interactions with actin and microtubules to ensure the integrity of the cytoskeleton machinery, depends on various factors operating at the transcriptional, translational, and post-translational levels [21]. EGFR governs the stability of the cytoskeleton through the MAPK/ERK pathway by phosphorylating Ser69 of the Bim isomers Bim_L_ and Bim_EL_ [39,40]. The phosphorylation of Bim isomers at Ser69 leads to ubiquitin-dependent or ubiquitin-independent 26S proteasome-mediated degradation of the protein in mice and rat apoptotic models [21]. The treatment of cancer cells with potent EGFR TKIs has been shown to increase Bim_L_ and Bim_EL_ expression levels [40,41]. In any case, the sequestration of Bim through a variety of signaling and metabolic pathways sensitizes the cytoskeleton to autophagy [42]. 

**Figure 8:**
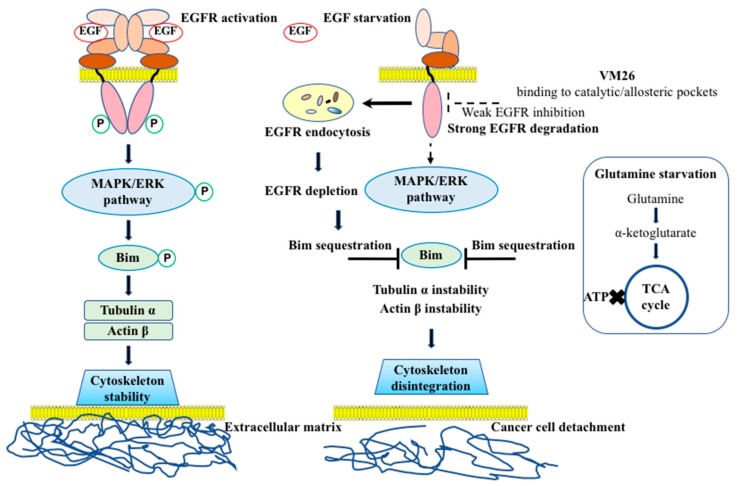
The proposed mechanism of the action of EGFR degraders on cancer cells. The binding of EGF to the extracellular region of the receptor monomers results in rearrangements favorable to the formation of functionally active homodimers that trigger downstream signaling pathways [1]. The compound VM26 binds to the tyrosine kinase catalytic pocket extended into the hydrophobic allosteric site inducing EGFR trafficking and degradation in endosomes (microautophagy). The depletion of EGFR results in Bim sequestration that leads to cytoskeleton disintegration, and ultimately to cancer cell detachment from the extracellular matrix. Glutamine starvation causes the deficiency of α-ketoglutarate and thereby, the inability of cells to replenish the tricarboxylic acid (TCA) cycle and produce ATP. Dual starvation for EGF and glutamine accelerates and reinforces the cytoskeleton disintegration leading ultimately to cancer cell detachment-promoted death.

Our study suggests a plausible mechanism of action of polyfunctional heterocyclic compounds and the course of events that cause Bim_EL_ sequestration-mediated degradation of the cytoskeleton, leading to cancer cell detachment-promoted death (Figure 8). The action of new compounds is phenotypically similar to the programmed apoptosis, anoikis, whereas the chemical invasion leads to the “forced death” that is different from a natural process. 

Binding of the most active compound, VM26, to EGFR enhances endocytic degradation and essential depletion of the receptor protein, leading to up-regulation but no phosphorylation of Bim_EL_. Therefore, the inactive sensor protein cannot provide the maintenance of functional relationships and the integrity of cytoskeleton proteins in cancer cells. The withdrawal of serum from the growth medium, which is the source of EGF [32] and competes with the active compound VM26 for binding to EGFR, accelerates the cytoskeleton degradation and detachment of cells from the ECM. 

However, glutamine starvation also plays a crucial role in strengthening protein degradation with VM26. The addition of the cognate ligand EGF to continuously FBS-starved culture in the absence of the compound weakly improved Bim_EL_ expression but not protein phosphorylation (Figure 6). Meanwhile, when only glutamine was added to the starvation culture, it increased the phosphorylation rate of Bim_EL_ in the absence of VM26 but not in the presence of the compound. Therefore, the expression and phosphorylation rate of Bim_EL_ upon the treatment with the compound VM26 is dynamic process that is modulated by the availability of both cognate ligand(s) and energy resources for EGFR activation. 

We questioned how glutamine starvation affects signaling pathways initiated by EGFR and why the deficiency in glutamine strengthens the potency of VM26 to kill cancer cells. First, cancer cells are fast-growing cells and require more energy in the form of ATP for protein synthesis than normal cells [43]. Second, the addition of glutamine to the growth medium is necessary for many tumor cell lines, not only because of the instability of this amino acid at 37 °C but also as a source of energy for cellular metabolism [44]. Third, glutamine is enzymatically converted into α-keto-glutarate, which merges with the TCA cycle to provide high levels of ATP production [45]. Fourth, EGFR requires ATP not only to activate the catalytic ATP-binding site but also to phosphorylate many amino acids, including seven tyrosine residues, even if the receptor undergoes endocytosis [46]. Fifth, the ATPase activity of the chaperone Hsp90α provides energy-dependent correction of a large number of misfolded client proteins to protect them against ubiquitination and 26S proteasome-promoted degradation [47]. As the levels of Hsp90α decrease with the depletion of EGFR (Figure 3 and Figure 5), misfolded glutamine-prone proteins become accessible to protease degradation. Therefore, the dependence of EGFR activation and related processes on glutamine can be formulated as “no glutamine, no EGFR signaling”. 

Glutamine starvation has been applied for cancer therapy [48]. In this regard, recent data have demonstrated that a potent TKI of EGFR, erlotinib, in combination with the glutaminase inhibitor CB-839 severely affects metabolic and energetic balances, leading to apoptotic death of NSCLC cancer cells [49]. The dual starvation for glutamine and EGF, resulting in the deficiency of ATP and the weakness of EGFR functions, accelerates and reinforces the autophagic protein degradation in cancer cells exposed to the compound VM26. Obviously, the rate of glutamine consumption primarily determines the fate of cells, which can be nuanced by the influence of other factors on reprogramming cellular processes, which may explain the variability in the time required for Bim sequestration and cytoskeleton disintegration observed in our experiments. Therefore, further clarification of small compound invasion in the modulation of EGFR signaling and glutamine metabolism axis is of undeniable interest towards improving treatment strategies for tumors.

Another important issue in our study is the identification of a putative molecular switch for EGFR endocytic degradation. The structure-activity relationship analysis strongly suggests that receptor degradation is a feature determined by carboxylic acid extremity. Indeed, in contrast to the compounds VM25 and VM26, two other compounds, VM20 and VM21—which have the same structure except for the ester group instead of the carboxylic acid (Figure 1)—were not able to promote degradation of EGFR under the conditions used (Figure 2a). In this regard, the presence of the carboxylate group in the furan moiety of compounds determines the accommodation of the hydrophobic alkyl-ether chain in the hydrophobic allosteric pocket located in the immediate vicinity of the ATP-binding site (Figure 7b,c). Furthermore, the cytotoxicity of small compounds correlates with protein degradation and cell-detachment abilities, showing the following order of efficacy: VM26 > VM25 > VM24 > VM23 > VM22. This order correlates with the length of the alkyl-ether chain in their structures, CH_3_(CH_2_)_4_, CH_3_(CH_2_)_3_, CH_3_(CH_2_)_2_, CH_3_CH_2_, and CH_3_, respectively (Figure 1). Therefore, the more the alkyl chain CH_3_(CH_2_)n occupies the hydrophobic allosteric pocket, the greater the EGFR degradation is, which correlates with increased detachment of cancer cells from the ECM and toxicity of compounds in breast cancer cells. 

One can postulate that the local hydrophobicity of the allosteric pocket, enhanced by the longer alkyl-ether chains in compounds, is used as a signal for proteases to attack the bound small compound-EGFR complexes in lysosomes. According to MD analysis, the reorientation of the compounds VM25 and VM26 from Arg803 to Arg841 is consistent with the participation of Arg841 in dynamic changes preceding the sulfenylation of Cys797 [9]. This structural rearrangement leads to the positioning and likely direct interaction of longer alkyl-ether chains with Met766 in the αCβ4 loop, which is located in the proximity to the EGFR ATP-binding site (Figure 7b) and is likely to be recognized by Hsp90α [50]. Structural changes caused by the compounds may also disrupt physical interaction of EGFR with Beclin 1, which, when phosphorylated, suppresses autophagy by modulating the Beclin 1 interactome [51]. In any case, the depletion of EGFR by small chemical degraders leads to the exclusion of the receptor from a global control of the integrity of the cytoskeletal machinery. 

The apoptotic death of cancer cells caused by chemical agents that enhance protein degradation has attracted increased attention in fighting cancer drug resistance [52]. The targeted degradation of proteins was first demonstrated by the construction of a ubiquitin ligase fusion protein [53]. The approach, known as PROTAC (Proteolysis Targeting Chimeric Molecule) [54], has been successfully used to specifically degrade protein kinases, including resistance to chemotherapy EGFR mutants by tagging potent inhibitors gefitinib, lapatinib or afatinib with a ubiquitination-sensitive module to sensitize the receptor protein to proteases [55]. Our independently performed research highlights that EGFR degradation can be alternatively and efficiently achieved with weak reversible EGFR inhibitors through the allosteric site, without conjugation of ubiquitin degradation-sensitive tags to potent TKIs. 

The chemotherapeutic interruption of MAPK/ERK phosphorylation flux has been considered a promising means to reduce metastatic dissemination and attenuate cancer progression [56]. In this regard, anti-EGFR inhibitors of the fourth generation demonstrate more selective binding to EGFR compared with other tyrosine kinase proteins, and this highlights a considerable advantage of allosteric inhibitors over catalytic site inhibitors in terms of therapeutic expectations [36,57]. According to our data, allosteric binders enhance EGFR degradation, which can largely compensate for weak inhibition of the catalytic site in breast cancer cells. The compound VM26 demonstrates high protein degradation in EGFR-positive prostate cancer, which appears to be naturally tolerant to anti-EGFR therapy in clinical studies [58]. In addition, the first in vivo assays showed promising results of our EGFR degraders in inhibiting the tumor growth in mice [59].

Inspired by these results, we put forward the hypothesis that targeting the allosteric site with weak reversible inhibitors, but potent degraders, can provide less brutal disruption of a variety of interconnected signaling and metabolic pathways, governed by EGFR, and lead to more selective killing cancer cells. This treatment may decrease secondary effects related to unpredicted aberrant consequences caused by potent TKIs in cancer and healthy cells. Therefore, targeting EGFR degradation through the drug-bound allosteric site in combination with glutamine starvation may be a rational and patient-friendly means for the treatment of EGFR-driven tumors.

## 4. Materials and Methods

### 4.1. Chemicals and Reagents

Gefitinib (ZD1839) and canertinib (CI-1033) were purchased from Sigma-Aldrich and Santa Cruz Biotechnology, respectively. Stock solutions of synthesized and commercial chemical compounds were prepared in DMSO (99.9% purity, OriGen Biomedical, Austin, TX, USA), and aliquots were stored at −80 °C. Tissue culture plastic wares (Nunc, Roskilde, Denmark) were used for growth of adherent cancer cells. Protease and phosphatase inhibitor cocktail tablets and anti-human anti-ErbB2 antibody were purchased from Thermo Fisher. Anti-phospho-EGFR (Tyr1068), anti-Hsp90α, and anti-HDAC2 antibodies were obtained from R and D Systems. Anti-EGFR (the epitope in the cytoplasmic region), LC3α/β, anti-LAMP-2, anti-β-actin, anti-Bim mouse monoclonal antibodies, fluorescent BP-CFL 595-labeled mIgGk, horseradish peroxidase-conjugated (HRP)-labeled mIgGk, and protein G-agarose were purchased from Santa Cruz Biotechnology (Heidelberg, Germany). Anti-EGFR mAb (the epitope in the extracellular region) was obtained from Millipore. Anti-phospho-Bim (Ser69) rabit mAb, HRP-labeled rabit mAb, and anti-phospho-Tyr (pTyr-100) mouse mAb conjugated to biotin were purchased from Cell Signaling Technology. HRP-labeled mouse mAb and WesternSure chemiluminescent substrate were obtained from Li-Cor (Lincoln, NE, USA). Chemical synthesis of compounds was described previously [29]. The chemistry of the compounds VM22-VM26 is described in Supplementary Text S1.

### 4.2. Cell Culture 

The triple-negative breast cancer MDA MB468 cells (obtained from Dr. Helene Hurst, Queen Mary University of London, UK) were grown in Dulbecco’s Modified Eagle’s Medium (DMEM) supplemented with 10% (*v*/*v*) foetal bovine serum (FBS), 2 mM L-glutamine, penicillin (100 units/mL), and streptomycin (100 µg/mL) in a humidified atmosphere of 5% CO_2_ in air. The non-small-cell lung-cancer A549 cells (obtained from the ATCC) and prostate cancer DU-145 cells (obtained from Dr. Fabrice Fleury, University of Nantes, France) were grown in RPMI-1640 supplemented with 5% FBS (for A549) or 10% FBS (for DU-145), 2 mM L-glutamine and corresponding antibiotics. To assess the action of the compounds in starved cultures, the cells were incubated in FBS-deprived media for 12–24 h. For stimulation of EGFR phosphorylation, compound-treated or nontreated cells were washed in PBS and incubated in FBS-deprived medium in the presence of EGF (200 ng/mL). The cells were washed two times in cold PBS, subsequently lysed in RIPA or Pierce IP lysis buffer supplemented with a cocktail of protease and phosphatase inhibitors, and centrifuged at 12,000 *g* for 10 min. The supernatant fraction was used for protein analyses. 

### 4.3. Cell Viability

Cell viability assays were performed with a colorimetric method [60] and IC50 values were calculated as an average of triplicate assays. 

### 4.4. Cell Detachment Assay

Cell growth plates that mimic the ECM (Nunc) were used for cultivation of cancer cells. Culture medium and 1× PBS-wash-off suspensions were carefully collected in one tube as a pool of putative detached cells. After centrifugation at 12,000 *g* for 5 min, the supernatant was discarded and the pellet was treated with lysis buffer. The rest of the cells attached to the well surface were separately lysed and collected by centrifugation in another tube. The ratio of lysis buffer used for the treatment of detached cells to attached cells in cultures grown in serum-deprived DMEM was 1:4 and for the treatment of detached cells to attached cells in cultures grown in serum-supplemented DMEM was 1:10. The relative efficiency of cellular detachment (%) was estimated as the ratio of individual protein levels in the detached cells to attached cells. Cell detachment was also visually controlled with optical microscopy.

### 4.5. Western Blotting 

Equal amounts of proteins or equal amounts of cells extracts were loaded onto the TGX gradient (4–18%) gels (Bio-Rad France, Marnes-La-Coquette, France), separated by sodium dodecyl sulfate-polyacrylamide electrophoresis (SDS-PAGE) and transferred onto a nitrocellulose membrane with a Trans-Blot Turbo system (Bio-Rad). The transferred proteins were probed with primary antibodies and then immunoreactive proteins were detected with HRP-conjugated secondary antibodies. Chemiluminescent detection of proteins and quantification of the intensity of protein bands was performed with a C-Digit scanner (Li-COR, Lincoln, NE, USA). Cell lysates pre-cleared with protein G-agarose were used to detect Bim and phosphorylated Bim in the gel. Other conditions were described previously [13]. Original images of Western blots are shown in Supplementary Figure S9.

### 4.6. siRNA Interference Knockdown of EGFR

Transfection of MDA MB468 cells with siRNA was performed with 60 pmol of human EGFR-specific duplex siRNA or scrambled siRNA (control) according to the manufacturer’s recommendations (Santa Cruz Biotechnology, Heidelberg, Germany). Transfected cells were allowed to recover in complete DMEM for 48 h and then starved in FBS-free DMEM for 18 h before exposure to compounds VM25 or VM26 for 2 h. The receptor silencing was approximately 40% with the EGFR siRNA compared with the cells transfected with a scrambled siRNA in two independent experiments. 

### 4.7. Confocal Immunofluorescence Microscopy

MDA MBT68 cells (5 × 10^4^ cells per well) were transferred into 8-well μ-Slides (Ibidi) and starved in FBS-deprived DMEM for 18 h, and then incubated with 2.5 µM of compounds for one hour at 37 °C in the same medium. The cells were washed twice with cold PBS, immediately fixed with 4% paraformaldehyde at room temperature for 10 min, permeabilized with 0.25% Triton X-100, and then incubated with relevant antibodies at 37 °C for 60 min. The plasma membrane was visualized in non-permeabilized cells anti-EGFR mAb (the epitope in the extracellular region) conjugated to Cy5.5 and prepared as described previously [61]. The anti-EGFR mAb conjugated to Cy5.5 was also used to detect the receptor protein in the permeabilized cells. Proteins LC3α/LC3β and Bim were visualized with corresponding mAbs followed by incubation with mIgGk BP-CFL 595 (dilution 1:500) at 37 °C for 60 min. To visualize the nucleus, cells were stained with DAPI (dilution 1:1000) for 15 min at room temperature. The cells were viewed with a laser-scanning confocal microscope (Nikon A1RSi) and a wide-field microscope (Nikon Eclipse E800). The images were recorded with NIS Element software (V4.40) and processed with ImageJ software (NIH v1.52). 

### 4.8. Molecular Docking

The X-ray crystal structure of the EGFR kinase domain in complex with a non-covalent derivative of pyrimido [4,5-b]azepine scaffold (PDB 3W32) [35], and with allosteric inhibitors EAI001 and EAI045 (PDB ID 2GS7) and (PDB ID 2JIV) [36] were used for docking studies with compounds VM22-VM26. Molecular models of EGFR and chemical compounds were created in PDB format using the ChemBioDraw Ultra 12.0 software package (http://software.informer.com/getfree-chembio3d-ultra-12.0/). Minimization of the free energy of the compounds was conducted with the MM2 program in the ChemBioDraw Ultra 12.0 software package. The analysis of the compound interactions with the EGFR catalytic domain was performed with the AutoDock Vina software package (http://vina.scripps.edu/index.html) as described by Trott and Olson [62]. Putative binding of small compounds to EGFR was generated with the program AutoDock Tools 1.5.6rc3. The highest scoring values of the nine conformations were calculated for each coupled ligand-protein interaction using the Vina scoring function. 

### 4.9. Molecular Dynamics

To examine the dynamics and stability of the small molecules interactions with the EGFR kinase domain [35] molecular dynamics simulations were employed using GROMACS 5.1 software [63]. Amber03 and GAFF force fields were applied for the proteins and small molecules, respectively [64]. Each EGFR complex with a small molecule was simulated for 500 ns in explicit water. Energy minimization was performed with the steepest-descent method until the maximum force was less than 1000.0 kJ/mol/nm. MD simulations were performed with 10-fs time steps, and electrostatics values were treated with a Coulomb cutoff of 11 Å. The dynamic systems were temperature-coupled using the Berendsen algorithm with a 1.0 ps time constant [65], and isotropic pressure coupling was employed using the Berendsen algorithm with a time constant of 1.1 ps. The results from the MD simulations were analyzed with Gromacs utilities using Visual Molecular Dynamics (VMD) software version 1.9.2 [66]. Calculations were performed on a Graphics Processing Unit accelerated cluster (Synsight, Evry, France). 

### 4.10. Statistical Information 

Statistical analysis was performed with GraphPad Prism V6 Software. For individual value plots, data displayed as mean ± standard error of the mean. P values for statistical analyses were obtained with Student’s *t* test.

## 5. Conclusions

This study points to an alternative possibility in the fight against aggressive cancer by targeting EGFR with furfuryl derivatives of 4-allyl-5-[2-(4-alkoxyphenyl)quinolin-4-yl]-4H-1,2,4-triazole-3-thiol. New compounds have a low ability to inhibit EGFR-mediated tyrosine kinase activity. However, binding to catalytic and allosteric sites located in close proximity to each other in the tyrosine binding domain, determines their ability to trigger rapid and enhanced endocytic degradation of the receptor protein, leading to disintegration of the cytoskeleton and, ultimately, to cancer cell detachment-promoted death. The course of these distinct events, which are linked through the Bim sequestration, depends on the growth conditions and functional interplay preceding the cell death. The implication of autophagy as a cytoprotective response to chemical invasion apparently explains the low toxicity of EGFR degraders.

Targeting EGFR degradation to kill cancer cells, with less destructive effects on normal cells, is an attractive and promising rationale for further investigations aiming to stop metastatic progression and to override the drug resistance in EGFR-driven tumours.

## Supplementary Materials

The following materials are available online at https://www.mdpi.com/2072-6694/11/8/1094/s1. Figure S1: Protein degradation in MDA-MB-468 and A549 cells after 18-h treatment with increasing concentrations of VM26 in FBS-deprived media, Figure S2. The enlarged image of Figure 3c. Immunofluorescence detection of LC3α/β in MDA-MB-468 cells exposed to 2.5 µM VM26 for 1 h, Figure S3. The enlarged image of Figure 4a–d. EGFR rapidly responds to the chemical invasion of small compounds in cancer cells, Figure S4: Kinetics of the viability of MDA-MB-468 and A549 cells in the FBS-supplemented media during incubation with the compounds VM25 and VM26 for 72 h, Figure S5. Protein expression and phosphorylation in DU-145 cells treated with compound VM26. (a) Western blot of proteins in attached and detached cells after exposure to 200 ng/ml EGF or 25 µM VM26 for one and three hours in serum-deprived RPMI-1640 medium. The detached cells were lysed on one-tenth of the volume buffer used for attached cells. (b) Estimation of relative levels of the detached cells to the attached cells was carried out with anti-LAMP-2 mAb. (c) Protein profiling in cells left untreated or treated simultaneously with 25 µM VM26 and EGF (200 ng/ml) or glutamine (2.0 mM), Figure S6: Time dependence of the RMSD monitored during the formation of the complexes between EGFR and compound (A) VM23, (B) VM25, (C) VM26 or (D) gefitinib. The RMSD monitored for the protein backbone in the absence of a small compound (E), Figure S7: 2D representation of different types of interactions between EGFR and compound VM23 (A), VM25 (B), VM26 (C) or gefitinib (D). Images come from the average geometries of the molecular dynamic simulations during the last 50 ns, Figure S8: Comparison EAI045 and VM26 docking in 3D-resolved EGFR structures 2GS7 and 2JIV. The hydrogen bond is shown in the form of green chain, Figure S9: Western blot original images, Table S1: Two-dimensional size of nucleus in MDA-MB-468 cells treated with vehicle (DMSO 0.1%) and compound VM26 were measured from images in Figure 4, Table S2: Toxicity of the compounds (IC50) in MDA-MB-468 breast cancer cells were measured after the grown in FBS-supplemented media for 72 h, Table S3: Binding parameters for compounds VM22-VM26 were estimated by docking with the EGFR kinase domain, Table S4: Docking of compounds VM23, VM25, and VM26 and amino acids in the EGFR kinase domain. The three best docked configurations (Conf) and corresponding ΔG values of the bound compound-protein are shown, Table S5: Distances between the small molecules and selected amino acids in the EGFR catalytic pocket. Distances between the sulfur atom in compounds VM23, VM25, VM26 and Cys797 (-SH) or Arg841 (-O) were measured using the average geometry of the bound structures in the catalytic domain. The azote in Gefitinib (no sulfur in its structure) was used for the estimation of the distance from Cys797, Table S6: The best-score docking of the reference molecule EAI045 and the compound VM26 in EGFR using 2GS7 and 2JIV structures. Amino acid interactions with the compounds through hydrogen bonds are shown in red, Table S7: Comparison of compounds EAI001, EAI045, and VM22-VM26 binding to human EGFR. Binding parameters were estimated by molecular docking in 3D- structures 2GS7 and 2JIV. EAI001 and EAI045 were used as reference molecules, Text S1: Characterization of compounds VM17-VM26, Video S1: Dynamic view of the superposition of small compounds bound to EGFR.
