# Supplementary material for: Targeting Degradation of EGFR through the Allosteric Site Leads to Cancer Cell Detachment-Promoted Death
Source: Cancers (Basel). 2019 Aug 1;11(8):1094. doi: 10.3390/cancers11081094 (PMC6721407; doi:10.3390/cancers11081094)
Supplement: Supplementary file 1 [file cancers-11-01094-s001.pdf]

Supplementary Materials: Targeting Degradation of EGFR through the Allosteric Site Leads to Cancer Cell Detachment-Promoted Death

Melkon Iradyan, Nina Iradyan, Philippe Hulin, Artur Hambardzumyan, Aram Gyulkhandanyan, Rodolphe Alves de Sousa, Assia Hessani, Christos Roussakis, Guillaume Bollot, Cyril Bauvais, Vehary Sakanyan

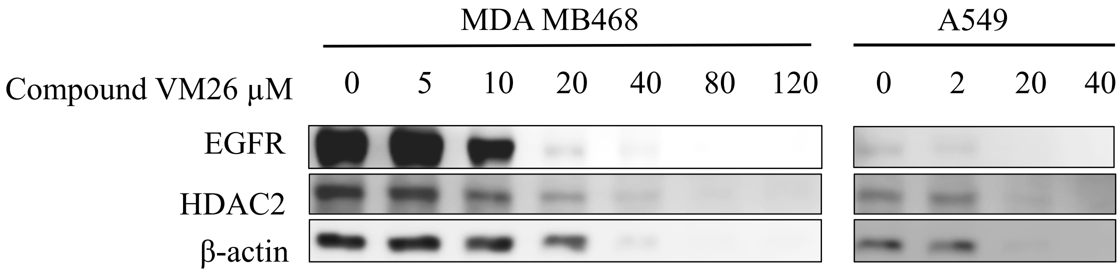

Figure S1. Protein degradation in MDA-MB-468 and A549 cells after 18-h treatment with increasing concentrations of VM26 in FBS-deprived media.

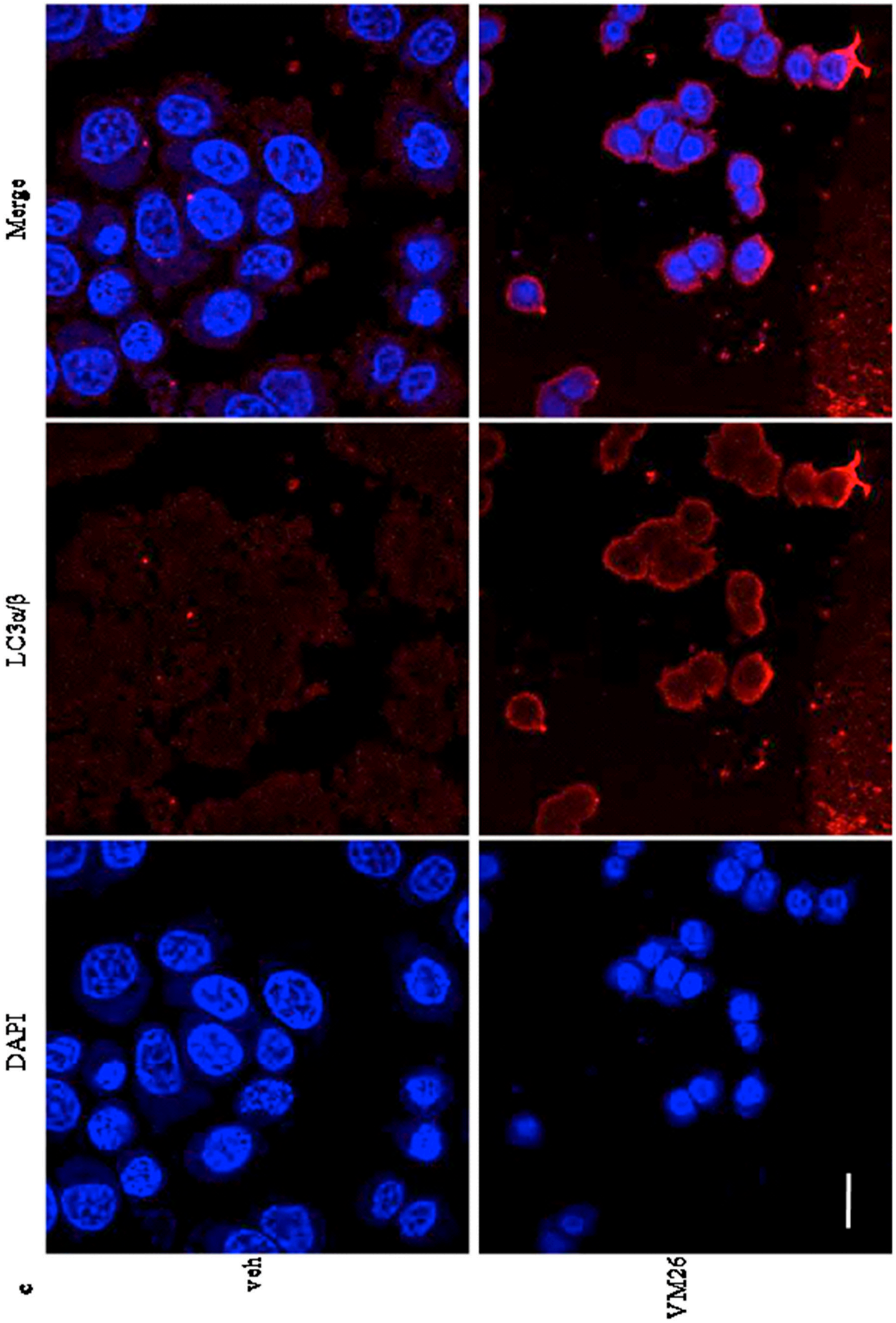

Figure S2. The enlarged image of Figure S3c. Immunofluorescence detection of LC3α/β in MDA-MB-468 cells exposed to 2.5 μM VM26 for 1 h. The size bar is equal to 10 μm.

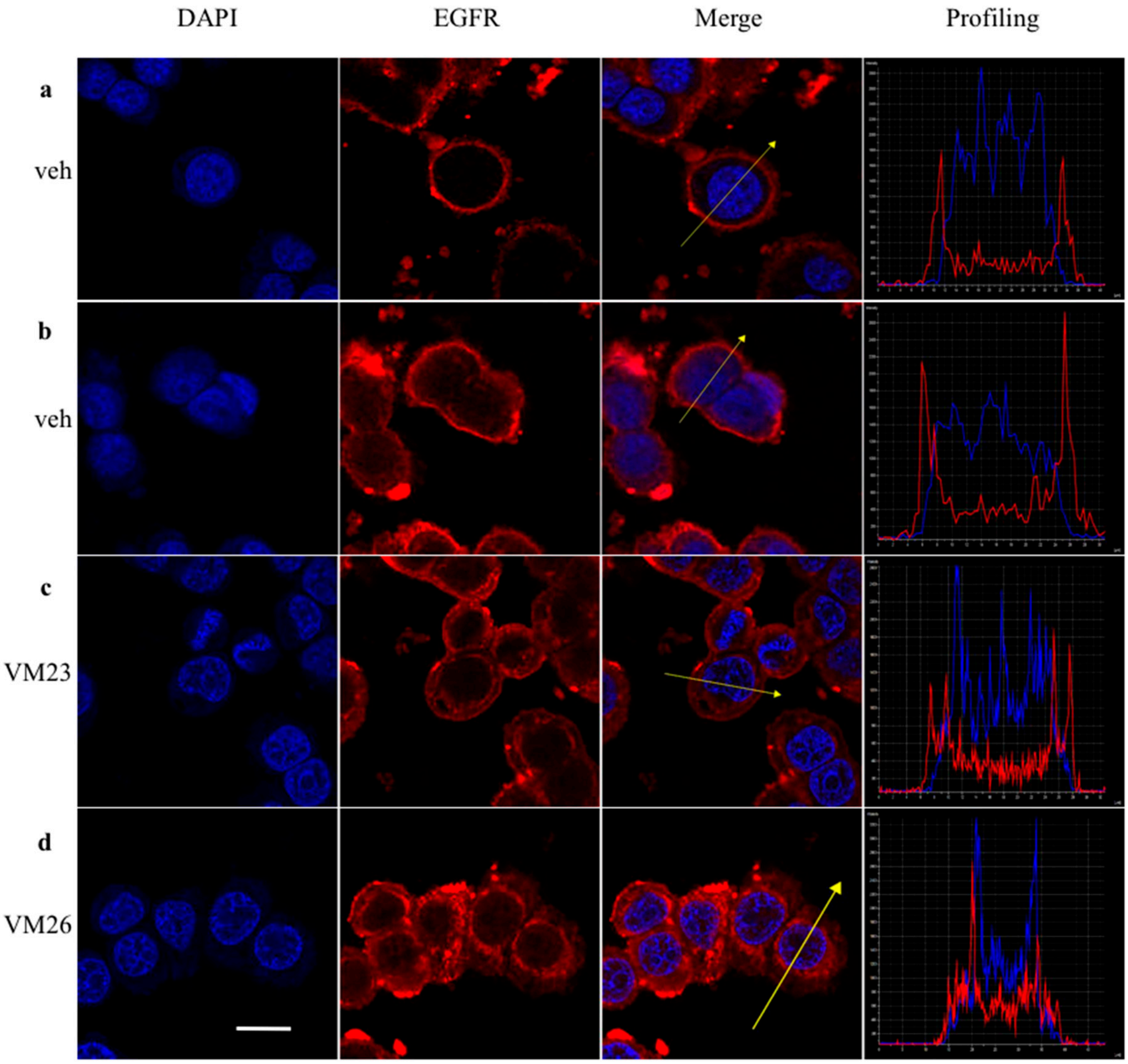

Figure S3. The enlarged image of Figure S4a, 4b, 4c, 4d. EGFR rapidly responds to the chemical invasion of small compounds in cancer cells. The size bar is equal to 10 μm.

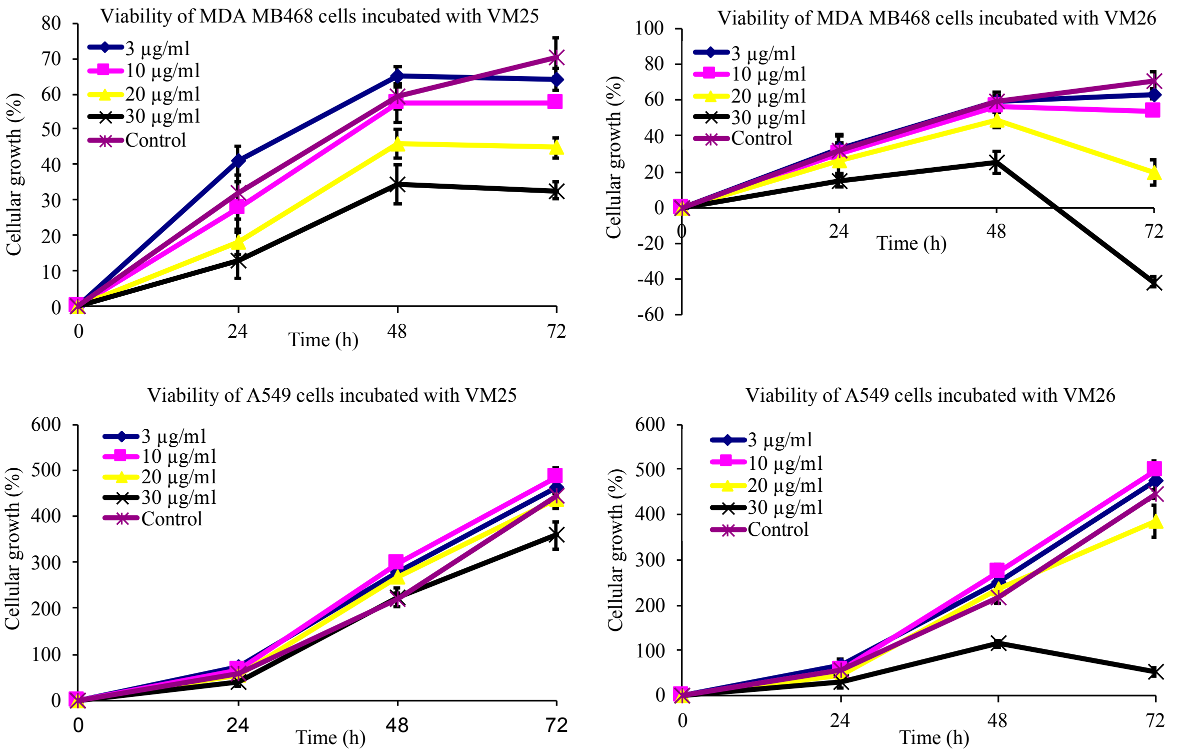

Figure S4. Kinetics of the viability of MDA-MB-468 and A549 cells in fetal bovine serum (FBS) supplemented media during incubation with the compounds VM25 and VM26 for 72 h.

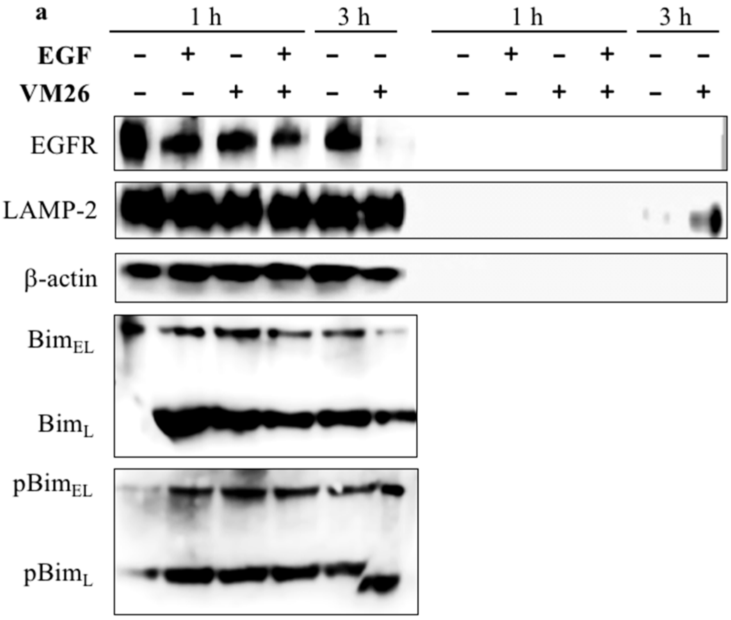

b

Time	Treatment	LAMP-2 Det/Att (%)
1 h	veh	0,09
	veh +EGF	0,01
	**VM26**	0,44
	**VM26** +EGF	0,17
3h	veh	1,32
	**VM26**	3,96

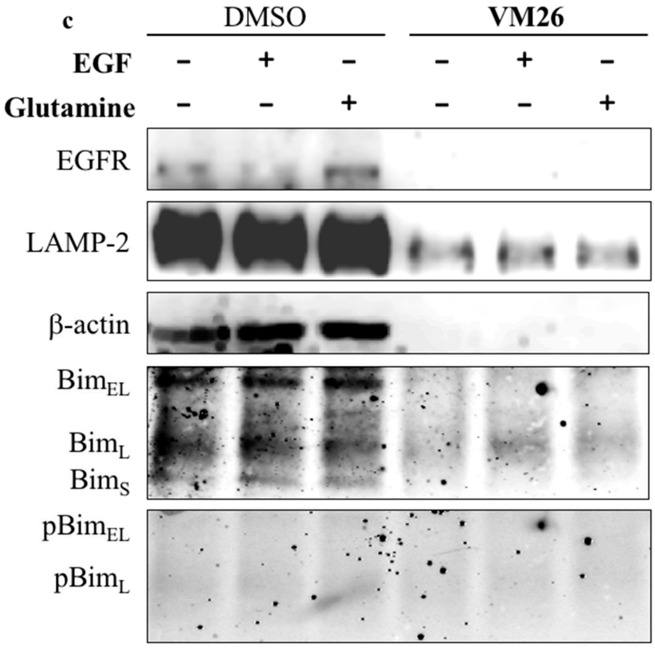

Figure S5. Protein expression and phosphorylation in DU-145 cells treated with compound VM26. (**a**) Western blot of proteins in attached and detached cells after exposure to 200 ng/ml EGF or 25 μM VM26 for one and three hours in serum-deprived RPMI-1640 medium. The detached cells were lysed on one-tenth of the volume buffer used for attached cells. (**b**) Estimation of relative levels of the detached cells to the attached cells was carried out with anti-LAMP-2 mAb. (**c**) Protein profiling in cells left untreated or treated simultaneously with 25 μM VM26 and EGF (200 ng/ml) or glutamine (2.0 mM).

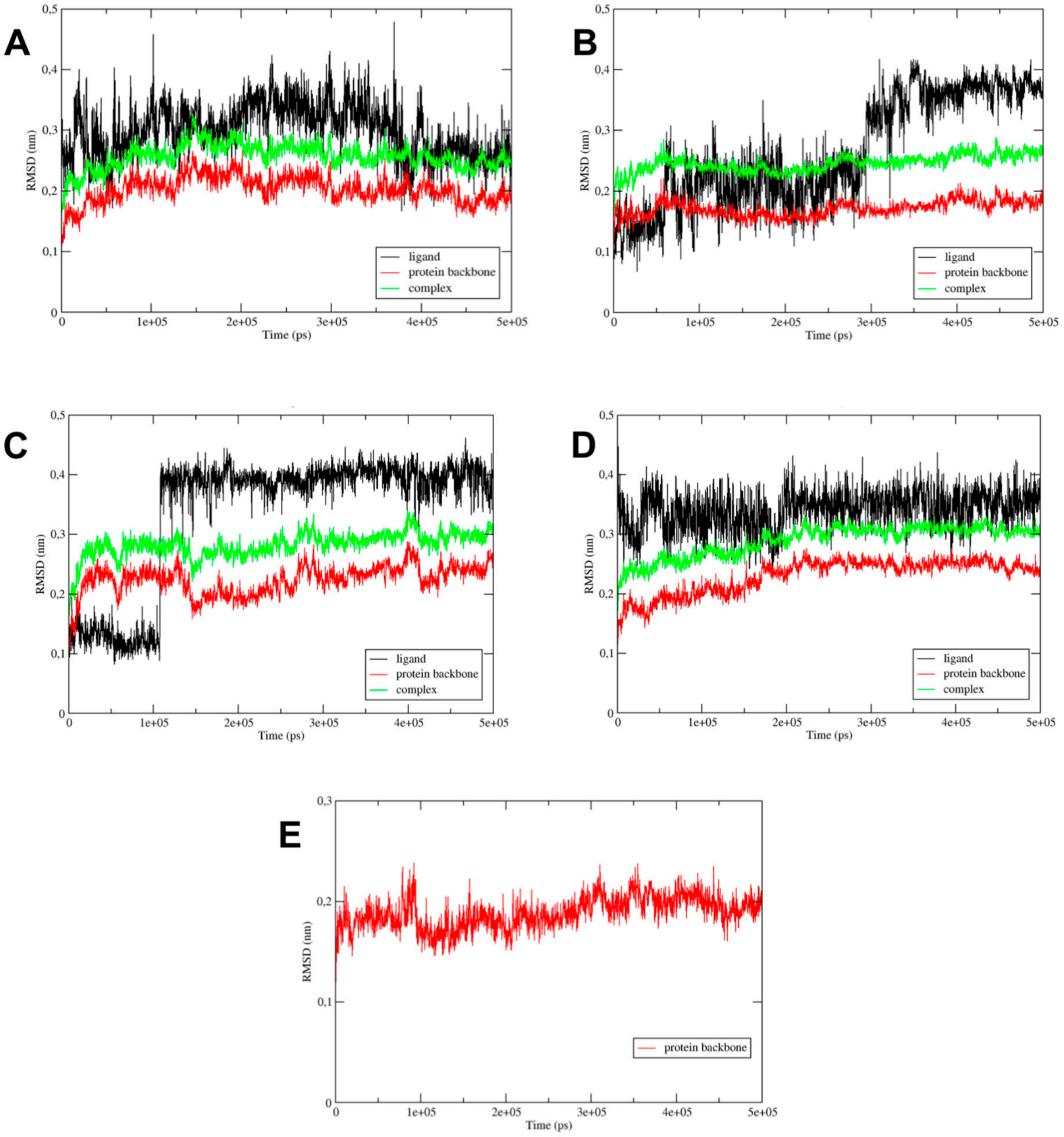

Figure S6. Time dependence of the RMSD monitored during the formation of the complexes between EGFR and compound (**A**) VM23, (**B**) VM25, (**C**) VM26 or (**D**) gefitinib. The RMSD monitored for the protein backbone in the absence of a small compound (**E**).

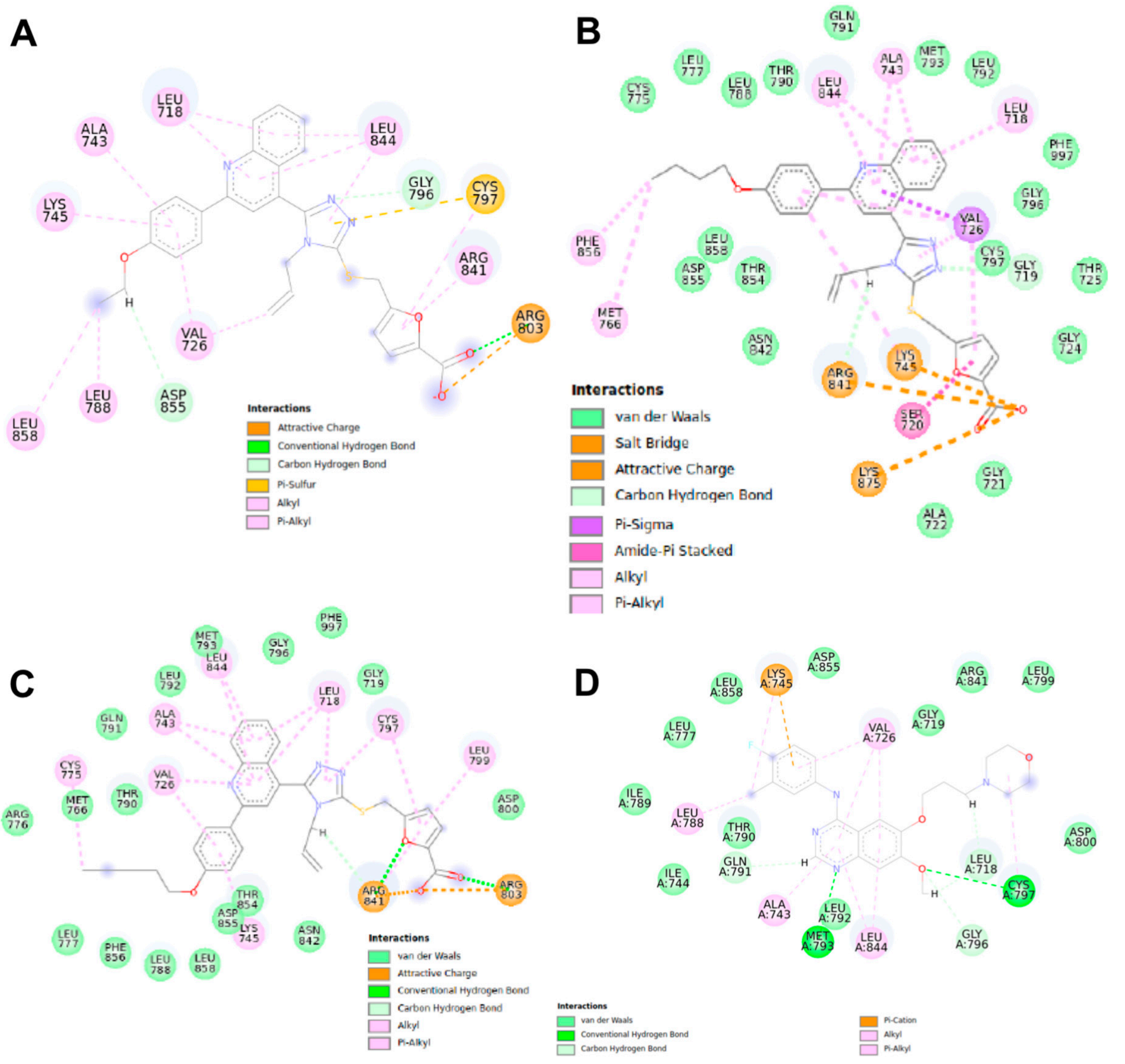

Figure S7. 2D representation of different types of interactions between EGFR and compound VM23 (**A**), VM25 (**B**), VM26 (**C**) or gefitinib (**D**). Images come from the average geometries of the molecular dynamic simulations during the last 50 ns.

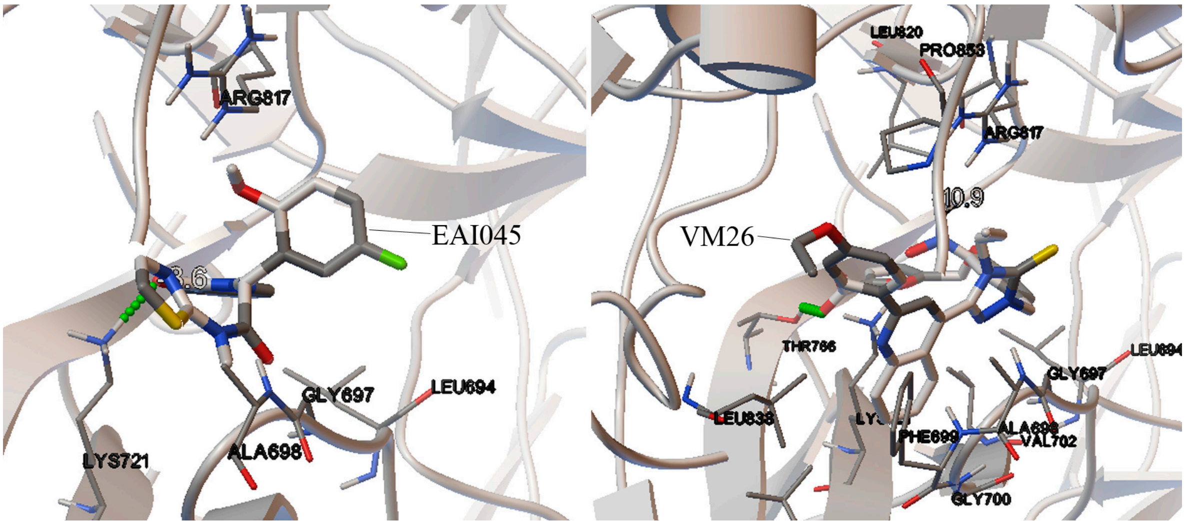

2GS7

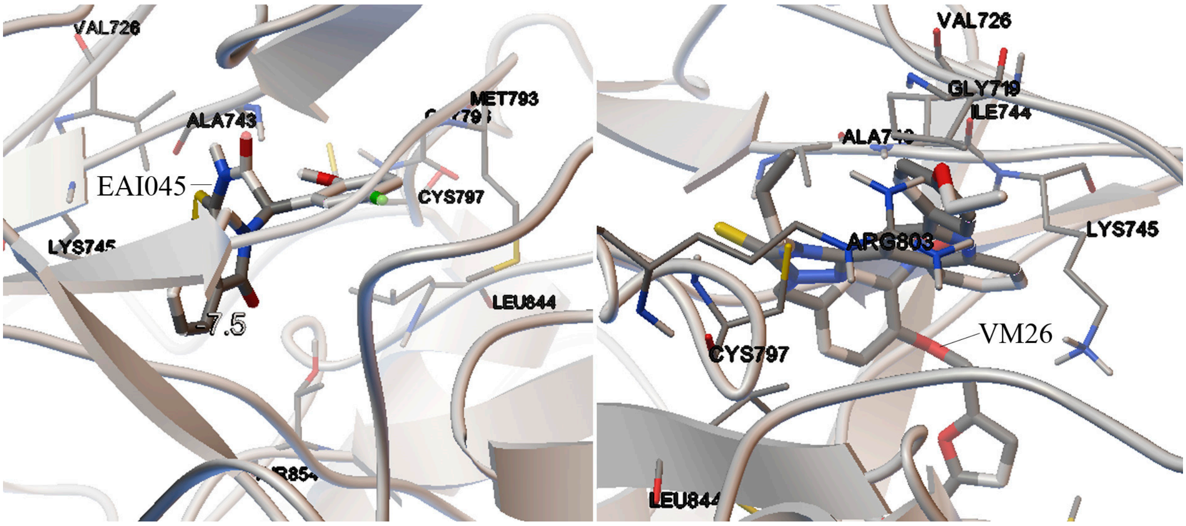

2JIV

Figure S8. Comparison EAI045 and VM26 docking in 3D-resolved EGFR structures 2GS7 and 2JIV. The hydrogen bond is shown in the form of green chain.

Fig. 2c WB original

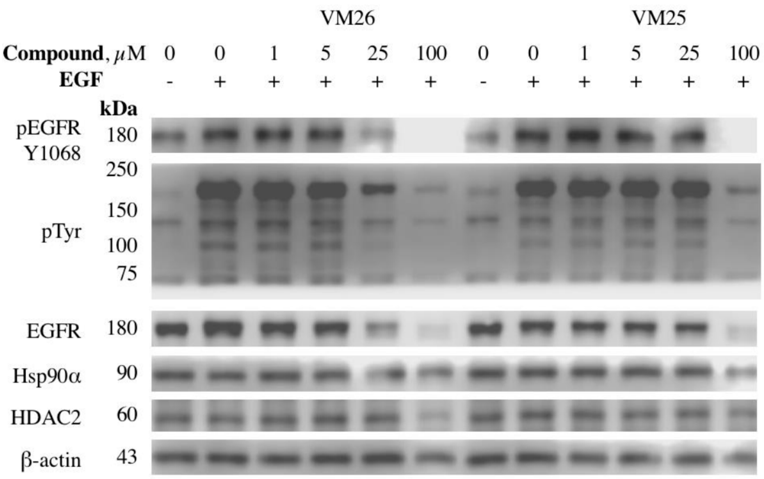

Fig. 3a WB original

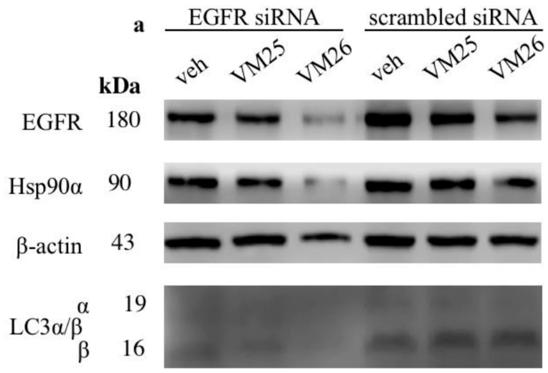

Fig. 5 WB original

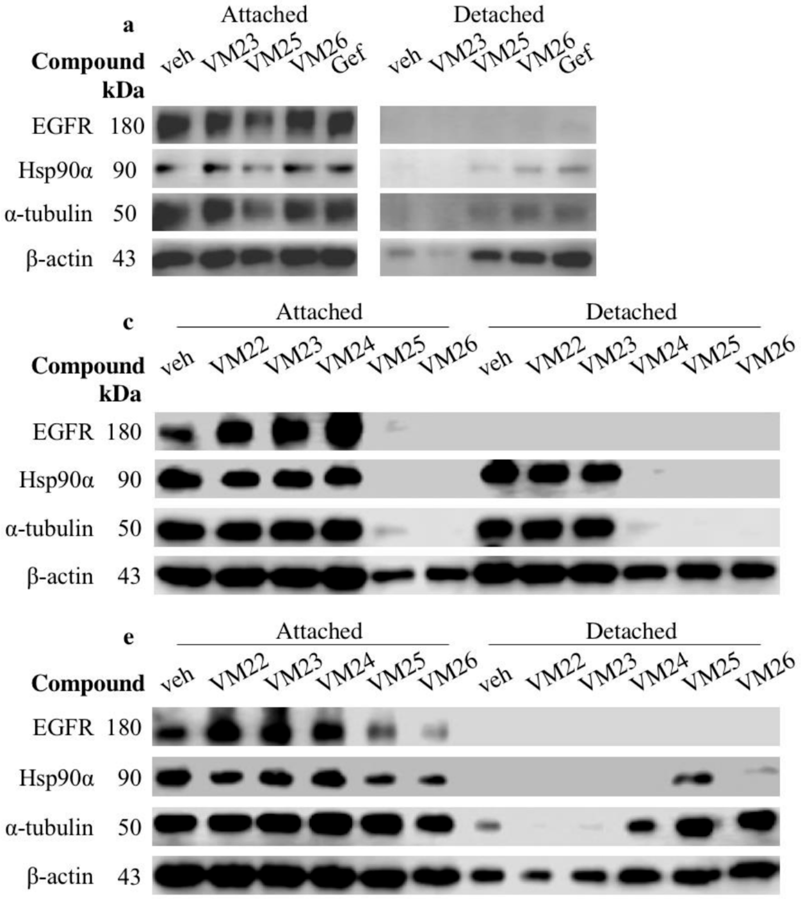

Fig. 6 WB original

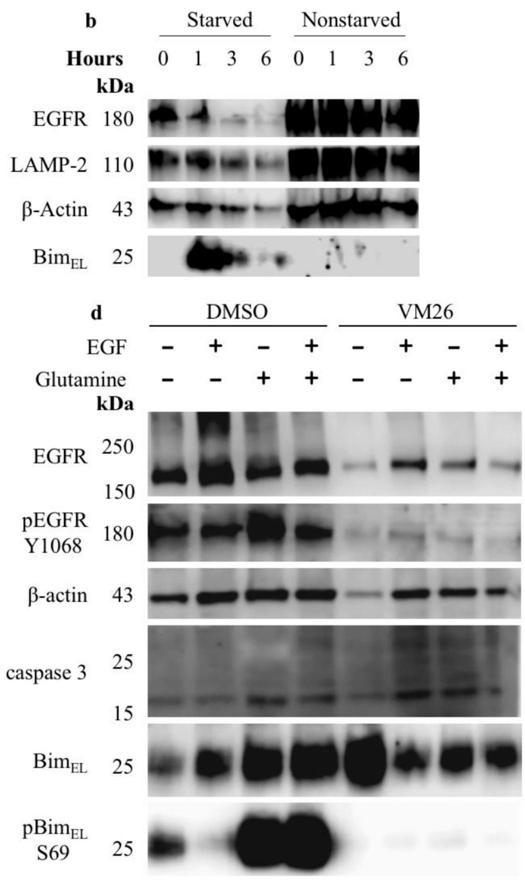

Fig. 7a WB original

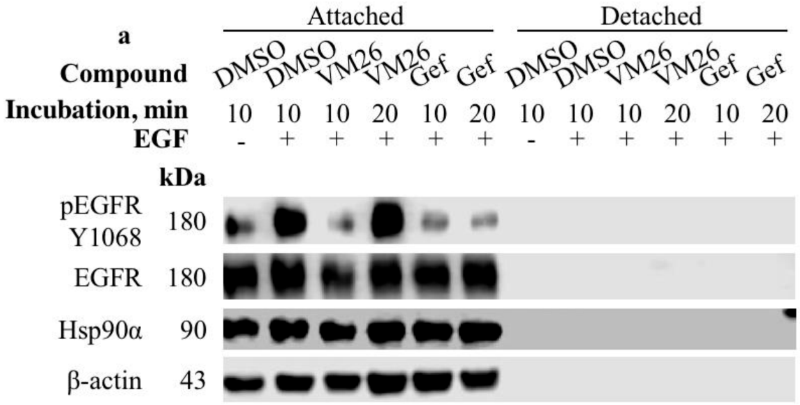

Supplementary Figure 9. Western blot original images.

Table S1. Two-dimensional size of nucleus in MDA-MB-468 cells treated with vehicle (DMSO 0,1%) and compound VM26 were measured from images in Figure S4.

**DMSO**	**119,408**	**VM26**	**108,706**
	111,681		93,785
	104,398		121,672
	156,22		130,198
	139,168		104,798
	162,348		58,971
	161,815		42,097
	135,038		114,567
	119,23		89,345
	120,606		89,522
	137,525		76,911
	193,343		127,889
	122,782		76,733
	113,59		87,924
	149,737		92,72
	167,854		90,766
	293,79		100,713
	342,281		111,015
	161,993		86,325
	233,93		120,962
	217,589		45,649
	183,663		81,707
	301,96		41,742
	147,072		79,398
	152,224		115,633
	178,689		127,179
	192,189		77,089
	205,511		71,227
	162,436		118,653
	152,712		116,876
			111,725
			117,232
			114,923
			98,048
			120,606
			86,147
			67,319
			110,66
			103,91
			137,125
			86,68
			43,695
			102,311
			106,397
			79,043
			87,924
			75,668
			79,398
			97,16
			71,76
			42,274
			80,464
			108,706
			44,761
			92,364

138,902
139,301
82,861
128,644
158,84
143,165
115,677
109,15
94,851
111,459

Table S2. Toxicity of the compounds (IC_{50}) in MDA-MB-468 breast cancer cells were measured after the grown in FBS-supplemented media for 72 h.

Compound	MDA-MB-468
VM22	> 60,0
VM23	47,9 ± 4,5
VM24	22,2 ± 2,7
VM25	14,4 ± 0,7
VM26	12,6 ± 1,8
gefitinib	15,2 ± 0,6

Table S3. Binding parameters for compounds VM22-VM26 were estimated by docking with the EGFR kinase domain.

Compound	ΔGo kcal/mol	K_D μM
VM22	−10.0	0.047
VM23	−9.9	0.055
VM24	−10.0	0.047
VM25	−10.2	0.033
VM26	−10.4	0.024
Gefitinib (ZD1839)	−9.0	0.253

Table S4. Docking of compounds VM23, VM25, and VM26 and amino acids in the EGFR kinase domain. The three best docked configurations (Conf) and corresponding ΔG values of the bound compound-protein are shown.

VM23/3W32			VM25/3W32			VM26/3W32		
**Conf. 1**	**Conf. 2**	**Conf. 3**	**Conf. 1**	**Conf. 2**	**Conf. 3**	**Conf. 1**	**Conf. 2**	**Conf. 3**
−9.9	−9.7	−9.7	−10.2	−10.1	−10.0	−10.4	−10.3	−10.0
		Leu718		Leu718			Leu718	
	Gly719	Gly719						
	Ser720	Ser720	Ser720		Ser720			Ser720
Gly721		Gly721	Gly721			Gly721		
Val726	Val726		Val726	Val726	Val726	Val726	Val726	Val726
	Ala743					Ala743		
Lys745	Lys745	Lys745		Lys745	Lys745	Lys745	Lys745	Lys745
						Met766		
						Leu777	Leu777	Leu777
				Leu788				
Leu792			Leu792	Leu792	Leu792	Leu792	Leu792	Leu792
	Met793	Met793			Met793	Met793		Met793

	Cys797			Cys797			Cys797	
Arg841			Arg841	Arg841	Arg841	Arg841	Arg841	Arg841
Asn842			Asn842		Asn842	Asn842		Asn842
	Leu844							
						Thr854		
			Asp855	Asp855		Asp855	Asp855	Asp855
					Phe856		Phe856	
				Phe997			Phe997	

Table S5. Distances between the small molecules and selected amino acids in the EGFR catalytic pocket. Distances between the sulfur atom in compounds VM23, VM25, VM26 and Cys797 (-SH) or Arg841 (-O) were measured using the average geometry of the bound structures in the catalytic domain. The azote in Gefitinib (no sulfur in its structure) was used for the estimation of the distance from Cys797.

Compound	Distance (Å) between compound and Cys797	Distance (Å) between compound and Arg841
VM23	5.04	4.71
VM25	4.74	3.42
VM26	3.67	3.17
Gefitinib	4.71	3.20

Table S6. The best-score docking of the reference molecule EAI045 and the compound VM26 in EGFR 2GS7 and 2JIV structures [1]. Amino acid interaction with the compounds through hydrogen bonds are shown in red.

EAI045/2GS7	VM26/2GS7	EAI045/2JIV	VM26/2JIV
G_o -8.6 kcal/mol	G_o -10.9 kcal/mol	G_o -7.5 kcal/mol	G_o -11.0 kcal/mol
A-Leu694	A-Leu694		
A-Gly697	A-Gly697		
A-Ala698	A-Ala698		
	A-Phe699		
	A-Gly700		
	A-Val702		
			A-Gly719
A-Lys721	A-Lys721		
	A-Leu723		
		B-Val726	A-Val726
		B-Ala743	A-Ala743
			A-Ile744
		B-Lys745	A-Lys745
			A-Cys775
	A-Thr766		A-Met766
		B-Met793	
		B-Gly796	
		B-Cys797	A-Cys797
			A-Arg803
A-Arg817	A-Arg817		
	A-Leu820		
	A-Leu838		
		B-Leu844	A-Leu844
	A-Pro853		
		B-Thr854	

Table S7. Comparison of compounds EAI001, EAI045, and VM22-VM26 binding to human EGFR. Binding parameters were estimated by molecular docking in 3D- structures 2GS7 and 2JIV [1]. EAI001 and EAI045 were used as reference molecules.

Ligand	2GS7		2JIV	
	G_o, kcal/mol	K_D, M	G_o, kcal/mol	K_D, M
EAI001	−8.6	0.4967	−9.1	0.2136
EAI045	−8.6	0.4967	−7.5	3.1800
VM22	−10.1	0.0395	−10.2	0.0334
VM23	−9.5	0.1087	−9.5	0.1087
VM24	−9.8	0.0655	−9.7	0.0776
VM25	−10.0	0.0468	−11.2	0.0062
VM26	−10.9	0.0102	−11.0	0.0086

Supplementary Text S1. Characterization of compounds VM17-VM26.

^{1}H NMR and ^{13}C NMR spectra were recorded in DMSO-d6/CCl_4 (1/3 v/v) at ambient temperature using a Varian Mercury-300 VX NMR spectrophotometer and chemical shifts were reported in ppm downfield from TMS. Electrospray ionization (ESI) and HRMS analyses were conducted using a Thermo Scientific Exactive Orbitrap spectrometer. The melting point was defined in °C on a Boecius micro-heating table. TLC was performed on Silufol UV-254 plates for compounds VM17-VM21 in 2:1 ethyl acetate-benzene, and for compounds VM22-VM26 in 10:2:1 ethyl acetate-methanol-water.

Methyl 5-{4-allyl-5-[2-(4-methoxyphenyl)quinolin-4-yl]-4H-1,2,4-triazole-3-ylsulphanyl-methyl}-2-furoate (VM17).

Yield 73 %, mp 103–104 °C. Rf 0.62. ^{1}H NMR, δ(ppm): 3.82 (s, 3H, OCH_3), 3.89 (s, 3H, OCH_3), 4.49 (dt, 2H, J = 5.1, 1.5 Hz, $CH_2CH{=}CH_2$), 4.62 (s, 2H, SCH_2), 4.81 (dq, 1H, J = 17.1, 1.5 Hz, $CH_2CH{=}CH_2$), 5.09 (dq, 1H, J = 10.5, 1.5 Hz, CH2CH=CH2), 5.73 (ddt, 1H, J = 17.1, 10.5, 5.1 Hz, $CH_2CH{=}CH_2$), 6.57 (d, 1H, J = 3.4 Hz, H-fur.), 6.99–7.05 (m, 2H, $C_6H_4OCH_3$), 7.12 (d, 1H, J = 3.4 Hz, H-fur.), 7.53 (ddd, 1H, J = 8.3, 6.9, 1.2 Hz, C_6H_4), 7.73–7.80 (m, 2H, C_6H_4), 7.73–7.80 (m, 2H, C_6H_4), 8.10 (s, 1H, =CH, pyr.), 8.12 (br.d, 1H, J = 8.3 Hz, C_6H_4), 8.21–8.26 (m, 2H, $C_6H_4OCH_3$). ^{13}C NMR, δ(ppm): 29.6 (SCH_2), 46.3 (NCH_2), 51.0 (OCH_3), 54.6 (OCH_3), 110.7 (=CH_2), 113,6 (2 CH), 117.5 (CH), 118.4 (CH), 118.8 (CH), 124.3, 124.8 (CH), 126.2 (CH), 128.3 (2 CH), 129.4 (CH), 129.5 (CH), 130.3, 131.3 (CH), 133.0, 143.4, 148.0, 149.7, 151.9, 154.3, 155.0, 157.5, 160.6. El. anal. calcd. for $C_{28}H_{24}N_4O_4S$: C 65.61; H 4.72; N 10.93; S 6.25; Found: C 65.49; H 4.66; N 10.77; S 6.06.

Methyl 5-{4-allyl-5-[2-(4-ethoxyphenyl)quinolin-4-yl]-4H-1,2,4-triazole-3-ylsulphanyl-methyl}-2-furoate (VM18).

Yield 82 %, mp 141–142 °C. Rf 0.65. ^{1}H NMR, δ(ppm) : 1.45 (t, 3H, J = 7.0 Hz, CH_3), 3.83 (s, 3H, OCH_3), 4.12 (q, 2H, J = 7.0 Hz, OCH_2), 4.49 (dt, 2H, J =5.1, 1.5 Hz, $CH_2CH{=}CH_2$), 4.62 (s, 2H, SCH_2), 4.81 (dq, 1H, J = 17.2, 1.5 Hz, $CH_2CH{=}CH_2$), 5.08 (dq, 1H, J = 10.4, 1.5 Hz, $CH_2CH{=}CH_2$), 5.73 (ddt, 1H, J = 17.2, 10.4, 5.1 Hz, $CH_2CH{=}CH_2$), 6.57 (d, 1H, J = 3.4 Hz, H-fur.), 6.97–7.02 (m, 2H, $C_6H_4OC_2H_5$), 7.12 (d, 1H, J = 3.4 Hz, H-fur.), 7.52 (ddd, 1H, J = 8.3, 6.9, 1.2 Hz, C_6H_4), 7.72–7.80 (m, 2H, C_6H_4), 8.09 (s, 1H, =CH, pyr.), 8.11 (br.d, 1H, J = 8.3 Hz, C_6H_4), 8.20–8.25 (m, 2H, $C_6H_4OC_2H_5$). ^{13}C NMR, δ(ppm): 14.3 (CH_3), 29.7 (SCH_2), 46.3 (NCH_2), 51.0 (OCH_3), 62.7 (OCH_2), 110.7 (=CH_2), 114.0 (2 CH), 117.5 (CH), 118.4 (CH), 118.8 (CH), 124.3, 124.8 (CH), 126.2 (CH), 128.3 (2 CH), 129.3 (CH), 129.4 (CH), 130.1, 131.3 (CH), 133.0, 143.4, 148.0, 149.7, 151.9, 154.3, 155.0, 157.5, 160.0. HRMS [ESI$^+$, MeOH]: calcd for $C_{29}H_{27}O_4N_4S$ $[M+H]^+$ 527.1748 found 527.1743. El. anal. calcd. for $C_{29}H_{26}N_4O_4S$: C 66.14; H 4.98; N 10.64; S 6.09; Found: C 66.10; H 4.83; N 10.72; S 6.05.

Methyl 5-{4-allyl-5-[2-(4-propoxyphenyl)quinolin-4-yl]-4H-1,2,4-triazole-3-ylsulphanyl-methyl}-2-furoate (VM19).

Yield 87 %, mp 130–131°C. Rf 0.72. ^{1}H NMR, δ(ppm): 1.09 (t, 3H, J = 7.4 Hz, CH_3), 1,78–1.91 (m, 2H, CH_2CH_3), 3.82 (s, 3H, OCH_3), 4.02 (t, 2H, J = 6.5 Hz, OCH_2), 4.48 (dt, 2H, J = 5.1, 1.5 Hz, CH_2CH=CH_2), 4.61 (s, 2H, SCH_2), 4.81 (dq, 1H, J = 17.1, 1.5 Hz, CH_2CH=CH_2), 5.08 (dq, 1H, J = 10.5, 1.5 Hz, CH_2CH=CH_2), 5.72 (ddt, 1H, J = 17.1, 10.5, 5.1 Hz, CH_2CH=CH_2), 6.57 (d, 1H, J = 3.5 Hz, H-fur.), 6.96–7.03 (2H, m, $C_6H_4OC_3H_7$), 7.12 (d, 1H, J = 3.5 Hz, H-fur.), 7.52 (ddd, 1H, J = 8.2, 7.0, 1.2 Hz, C_6H_4), 7.73–7.79 (m, 2H, C_6H_4), 8.09 (s, 1H, =CH, pyr.), 8.09–8.13 (m, 1H, C_6H_4), 8.20–8.25 (m, 2H, $C_6H_4OC_3H_7$). ^{13}C NMR, δ(ppm): 10.1 (CH_3), 21.9 (CH_2), 29.6 (SCH_2), 46.3 (NCH_2), 51.0 (OCH_3), 68.7 (OCH_2), 110.7 (=CH_2), 114.0 (2 CH), 117.5 (CH), 118.4 (CH), 118.8 (CH), 124.3, 124.8 (CH), 126.2 (CH), 128.3 (2 CH), 129.3 (CH), 129.4 (CH), 130.1, 131.3 (CH), 133.0, 143.4, 148.0, 149.6, 151.9, 154.3, 155.0, 157.5, 160.1. El. anal. calcd. for $C_{30}H_{28}N_4O_4S$: C 66.65; H 5.22; N 10.36; S 5.93; Found: C 66.57; H 5.10; N 10.51; S 5.75.

Methyl 5-{4-allyl-5-[2-(4-butoxyphenyl)quinolin-4-yl]-4H-1,2,4-triazole-3-ylsulphanyl-methyl}-2-furoate (VM20).

Yield 77 %, mp 115–116 0C. Rf 0.73. ^{1}H NMR, δ(ppm): 1.02 (t, 3H, J = 7.3 Hz, CH_3), 1.48-1.60 (m, 2H, CH_2CH_3), 1.75–1.85 (m, 2H, OCH_2CH_2), 3.82 (s, 3H, OCH_3), 4.05 (t, 2H, J = 6.4 Hz, OCH_2), 4.48 (br.d, 2H, J = 5.1 Hz, CH_2CH=CH_2), 4.62 (s, 2H, SCH_2), 4.81 (br.d, 1H, J = 17.2 Hz, CH_2CH=CH_2), 5.08 (br.d, 1H, J = 10.4 Hz, CH_2CH=CH_2), 5.72 (ddt, 1H, J = 17.2, 10.4, 5.1 Hz, CH_2CH=CH_2), 6.57 (d, 1H, J = 3.4 Hz, H-fur.), 6.97–7.02 (m, 2H, $C_6H_4OC_4H_9$), 7.12 (d, 1H, J = 3.4 Hz, H-fur.), 7.52 (ddd, 1H, J = 8.3, 7.0, 1.0 Hz, C_6H_4), 7.73–7.79 (m, 2H, C_6H_4), 8.09 (s, 1H, =CH, pyr.), 8.11 (br.d, 1H, J = 8.3 Hz, C_6H_4), 8.20–8.25 (m, 2H, $C_6H_4OC_4H_9$). ^{13}C NMR, δ(ppm): 13.4 (CH_3), 18.6 (CH_2), 29.6 (SCH_2), 30.7 (CH_2), 46.3 (NCH_2), 51.0 (OCH_3), 66.9 (OCH_2), 110.7 (=CH_2), 114.0 (2 CH), 117.5 (CH), 118.4 (CH), 118.8 (CH), 124.3, 124.8 (CH), 126.1 (CH), 128.3 (2 CH), 129.3 (CH), 129.4 (CH), 130.1, 131.3 (CH), 133.0, 143.4, 148.0, 149.6, 151.9, 154.3, 155.0, 157.5, 160.1. HRMS [ESI$^+$, MeOH]: calcd for $C_{31}H_{31}N_4O_4S$ $[M+H]^+$ 555,2061 found 555,2070. El. anal. calcd. for $C_{31}H_{30}N_4O_4S$: C 67.13; H 5.45; N 10.10; S 5.78; Found: C 67.05; H 5.31; N 10.26; S 5.94.

Methyl 5-{4-allyl-5-[2-(4-pentyloxyphenyl)quinolin-4-yl]-4H-1,2,4-triazole-3-ylsulphanyl-methyl}-2-furoate (VM21).

Yield 83 %, mp 91–92°C. Rf 0.76. ^{1}H NMR, δ(ppm): 0.97 (t, 3H, J = 7.1 Hz, CH_3), 1.34–1.55 (m, 4H, $CH_2CH_2CH_3$), 1.77–1.87 (m, 2H, OCH_2CH_2), 3.82 (s, 3H, OCH_3), 4.04 (t, 2H, J = 6.4 Hz, OCH_2), 4.48 (br.d, 2H, J = 5.0 Hz, CH_2CH=CH_2), 4.62 (s, 2H, SCH_2), 4.82 (br.d, 1H, J = 17.2 Hz, CH_2CH=CH_2), 5.09 (br.d, 1H, J = 10.4 Hz, CH_2CH=CH_2), 5.72 (ddt, 1H, J = 17.2, 10.4, 5.0 Hz, CH_2CH=CH_2), 6.57 (d, 1H, J = 3.4 Hz, H-fur.), 6.96–7.02 (m, 2H, $C_6H_4OC_5H_{11}$), 7.12 (d, 1H, J = 3.4 Hz, H-fur.), 7.52 (ddd, 1H, J = 8.3, 6.9, 1.1 Hz, C_6H_4), 7.73–7.79 (m, 2H, C_6H_4), 8.09 (s, 1H, =CH, pyr.), 8.11 (dd, 1H, J = 8.3, 1.5 Hz, C_6H_4), 8.20–8.25 (m, 2H, $C_6H_4OC_5H_{11}$). ^{13}C NMR, δ(ppm): 13.6 (CH_3), 21.8 (CH_2), 27.6 (CH_2), 28.3 (CH_2), 29.6 (SCH_2), 46.3 (NCH_2), 51.0 (OCH_3), 67.2 (OCH_2), 110.7 (=CH_2), 114.0 (2 CH), 117.5 (CH), 118.4 (CH), 118.8 (CH), 124.3, 124.8 (CH), 126.2 (CH), 128.3 (2 CH), 129.3 (CH), 129.4 (CH), 130.1, 131.3 (CH), 133.0, 143.4,148.0, 149.7, 151.9, 154.3, 155.0, 157.5, 160.1. HRMS [ESI$^+$, MeOH]: calcd for $C_{32}H_{33}N_4O_4S$ $[M+H]^+$ 569,2217 found 569,2229. El. anal. calcd. for $C_{32}H_{32}N_4O_4S$: C 67.58; H 5.67; N 9.85; S 5.64; Found: C 67.47; H 5.46; N 10.11; S 5.77.

5-{4-Allyl-5-[2-(4-methoxyphenyl)quinolin-4-yl]-4H-1,2,4-triazole-3-ylsulphanylmethyl}-furan-2-carboxylic acid (VM22).

Yield 82 %, mp 150–152 °C. Rf 0.65. ^{1}H NMR, δ(ppm): 3.88 (s, 3H, OCH_3), 4.48 (dt, 2H, *J* = 4.9, 1.5 Hz, $\underline{CH_2}CH$=CH_2), 4.59 (s, 2H, SCH_2), 4.80 (dq, 1H, *J* = 17.2, 1.5 Hz, CH_2CH=$\underline{CH_2}$), 5.08 (dq, 1H, *J* = 10.4, 1.5 Hz, CH_2CH=$\underline{CH_2}$), 5.72 (ddt, 1H, *J* = 17.2, 10.4, 1.5 Hz, $CH_2\underline{CH}$=CH_2), 6.51 (d, 1H, *J* = 3.3 Hz, H-fur.), 7.00–7.06 (m, 3H, $C_6H_4OCH_3$), 7.05(d, 1H, *J* =3.3 Hz, H-fur.), 7.51–7.57 (m, 1H, C_6H_4), 7.72–7.79 (m, 2H, C_6H_4), 8.09 (s, 1H, =CH, pyr.), 8.11 (dd, 1H, *J* = 8.6, 1.0 Hz, C_6H_4), 8.21–8.26 (m, 2H, $C_6H_4OCH_3$), 12 (br, 1H, COOH). ^{13}C NMR, δ(ppm): 29.9 (SCH_2), 46.3 (NCH_2), 54.7 (OCH_3), 110.6 (=CH_2), 113.7 (2 CH), 117.5 (CH), 117.8 (CH), 118.9 (CH), 124.4, 124 (CH), 126.3 (CH), 128.4 (2 CH), 129.4 (CH), 129.5

(CH), 130.4, 131.4 (CH), 133.1, 144.9, 148.1, 149.7, 152.0, 153.6, 155.0, 158.7, 160.6. HRMS [ESI$^+$, MeOH]: calcd for $C_{27}H_{23}O_4N_4S$ $[M+H]^+$ 499.1435, found 499.1441. El. anal. calcd. for $C_{27}H_{22}N_4O_4S$: C 65.04; H 4.45; N 11.24; S 6.43; Found: C 65.17; H 4.39; N 11.13; S 6.27.

5-{4-Allyl-5-[2-(4-ethoxyphenyl)quinolin-4-yl]-4H-1,2,4-triazole-3-ylsulphanylmethyl}-furan-2-carboxylic acid (VM23).

Yield 80 %, mp 156–157 °C. Rf 0.68. ^{1}H NMR, δ(ppm): 1.45 (t, 3H, *J* = 7.0 Hz, CH_3), 4.13 (q, 2H, *J* = 7.0 Hz, OCH_2), 4.48 (dt, 2H, *J* = 5.0, 1.5 Hz, $\underline{CH_2}CH{=}CH_2$), 4.60 (s, 2H, SCH_2), 4.80 (dq, 1H, *J* = 17.0, 1.5 Hz, $CH_2CH{=}\underline{CH_2}$), 5.08 (dq,1H, *J* = 10.4, 1.5 Hz, $CH_2CH{=}\underline{CH_2}$), 5.72 (ddt, 1H, *J* = 17.0, 10.4, 5.0 Hz, $CH_2\underline{CH}{=}CH_2$), 6.51 (d, 1H, *J* = 3.4 Hz, H-fur.), 6.98–7.03 (m, 2H, $C_6H_4OC_2H_5$), 7.05 (d, 1H, *J* = 3.4 Hz, H-fur.), 7.50–7.57 (m, 1H, C_6H_4), 7.72–7.79 (m, 2H, C_6H_4), 8.08 (s, 1H, =CH, pyr.), 8.11 (dd, 1H, *J* = 8.8, 1.0 Hz, C_6H_4), 8.19–8.25 (m, 2H, $C_6H_4OC_2H_5$), 12.50 (br, 1H, COOH). ^{13}C NMR, δ(ppm): 14.4 (CH_3), 29.9 (SCH_2), 46.3 (NCH_2), 62.8 (OCH_2), 110.6 (=CH_2), 114.1 (2 CH), 117.5 (CH), 117.8 (CH), 118.9 (CH), 124.3, 124.9 (CH), 126.3 (CH), 128.4 (2 CH), 129.4 (CH), 129.5 (CH), 130.2, 131.4 (CH), 133.1, 144.8, 148.1, 149.7, 152.0, 153.6, 155.1, 158.7, 160.0. HRMS [ESI$^+$, MeOH]: calcd for $C_{28}H_{25}O_4N_4S$ $[M+H]^+$ 513.1591, found 513.1590. El. anal. calcd. for $C_{28}H_{24}N_4O_4S$: C 65.61; H 4.72; N 10.93; S 6.26; Found: C 65.54; H 4.66; N 10.85; S 6.07.

5-{4-Allyl-5-[2-(4-propoxyphenyl)quinolin-4-yl]-4H-1,2,4-triazole-3-ylsulphanylmethyl}-furan-2-carboxylic acid (VM24).

Yield 86 %, mp 149–150°C. Rf 0.62. ^{1}H NMR, δ(ppm): 1.09 (t, 3H, *J* = 7.4 Hz, CH_3), 1.78–1.90 (m, 2H, $\underline{CH_2}CH_3$), 4.02 (t, 2H, *J* = 6.4 Hz, OCH_2), 4.48 (br.d, 2H, *J* = 5.0 Hz, $\underline{CH_2}CH{=}CH_2$), 4.60 (s, 2H, SCH_2), 4.80 (br.d, 1H, *J* =17.2 Hz, $CH_2CH{=}\underline{CH_2}$), 5.08 (br.d, 1H, *J* = 10.4 Hz, $CH_2CH{=}\underline{CH_2}$), 5.72 (ddt, 1H, *J* =17.2, 10.4, 5.0 Hz, $CH_2\underline{CH}{=}CH_2$), 6.51 (d, 1H, *J* =3.4 Hz, H-fur.), 6.98–7.03 (m, 2H, $C_6H_4OC_3H_7$), 7.04 (d, 1H, *J* = 3.4 Hz, H-fur.), 7.53 (ddd, 1H, *J* = 8.3, 7.0, 1.2 Hz, C_6H_4), 7,73–7.79 (m, 2H, C_6H_4), 8.08 (s, 1H, =CH, pyr.), 8.11 (br.d, 1H, *J* = 8.3 Hz, C_6H_4), 8.19–8.24 (m, 2H, $C_6H_4OC_3H_7$), 12.50 (br, 1H, COOH). ^{13}C NMR, δ(ppm): 10.2 (CH_3), 22.0 (CH_2), 29.9 (SCH_2), 46.3 (NCH_2), 68.8 (OCH_2), 110.6 (=CH_2), 114.2 (2 CH), 117.5 (CH), 117.7 (CH), 118.9 (CH), 124.3, 124.9 (CH), 126.3 (CH), 128.4 (2 CH), 129.4 (CH), 129.5 (CH), 130.2, 131.4 CH), 133.1, 144.8, 148.1, 149.7, 152.0, 153.6, 155.0, 158.7, 160,2. HRMS [ESI$^+$, MeOH]: calcd for $C_{29}H_{27}O_4N_4S$ $[M+H]^+$ 527.1748, found 527.1753. El. anal. calcd. for $C_{29}H_{26}N_4O_4S$: C 66.14; H 4.98; N 10.64; S 6.09; Found: C 66.03; H 4.75; N 10.80; S 6.17.

5-{4-Allyl-5-[2-(4-butoxyphenyl)quinolin-4-yl]-4H-1,2,4-triazole-3-ylsulphanylmethyl}-furan-2-carboxylic acid (VM25).

Yield 81 %, mp 158–159°C. Rf 0.70. ^{1}H NMR, δ(ppm): 1.02 (t, 3H, *J* = 7.3 Hz, CH_3), 1.48–1.61 (m, 2H, $\underline{CH_2}CH_3$), 1.75–1.85 (m, 2H, $\underline{CH_2}CH_2O$), 4.05 (t, 2H, *J* = 6.4 Hz, OCH_2), 4.48 (dt, 2H, *J* =5.0, 1.7 Hz, $\underline{CH_2}CH{=}CH_2$), 4.60 (s, 2H, SCH_2), 4.80 (dq, 1H, *J* = 17.1, 1.7 Hz, $CH_2CH{=}\underline{CH_2}$), 5.08 (dq, 1H, *J* = 10.4, 1.7 Hz, $CH_2CH{=}\underline{CH_2}$), 5.72 (ddt, 1H, *J* = 17.1, 10.4, 5.0 Hz, $CH_2\underline{CH}{=}CH_2$), 6.52 (d, 1H, *J* = 3.4 Hz, H-fur), 6.97–7.03 (m, 2H, $C_6H_4OC_4H_9$), 7.05 (d, 1H, *J* = 3.4 Hz, H-fur), 7.53 (ddd, 1H, *J* =8.2, 6.7, 1.2 Hz, C_6H_4), 7.73–7.79 (m, 2H, C_6H_4), 8,08 (s, 1H, =CH, pyr.), 8.11 (dd, 1H, *J* =8.7, 1.2 Hz, C_6H_4), 8.19–8.24 (m, 2H, $C_6H_4OC_4H_9$), 12.70 (br, 1H, COOH). ^{13}C NMR, δ(ppm): 13.5 (CH_3), 18.7 (CH_2), 29.9 (SCH_2), 30.7 (CH_2), 46.3 (NCH_2), 66.9 (OCH_2), 110.6 (=CH_2), 114.1 (2 CH), 117.5 (CH), 117.7 (CH), 118.8 (CH), 124.3, 124.9 (CH), 126.3 (CH), 128.4 (2 CH), 129.3 (CH), 129.5 (CH), 130.2, 131.4 (CH), 133.1, 144.8, 148.0, 149.7, 152.0, 153.6, 155.0, 158.7, 160.1. HRMS [ESI$^+$, MeOH]: calcd for $C_{30}H_{29}O_4N_4S$ $[M+H]^+$ 541.1904, found 541.1915. El. anal. calcd. for $C_{30}H_{28}N_4O_4S$: C 66.65; H 5.22; N 10.36; S 5.93; Found: C 66.52; H 5.10; N 10.23; S 6.07.

5-{4-Allyl-5-[2-(4-pentyloxyphenyl)quinolin-4-yl]-4H-1,2,4-triazole-3-ylsulphanylme-thyl}-furan-2-carboxylic acid (VM26).

Yield 89 %, mp 165–166°C. Rf 0.69. ^{1}H NMR, δ(ppm): 0.97 (t, 3H, *J* = 7.1 Hz, CH_3), 1.36–1.55 (m, 4H, $\underline{CH_2}\underline{CH_2}CH_3$), 1.76–1.86 (m, 2H, $OCH_2\underline{CH_2}$), 4.04 (t, 2H, *J* = 6.5 Hz, OCH_2), 4.48 (dt, 2H, *J* = 5.1, 1.5

Hz, CH$_2$CH=CH$_2$), 4.60 (s, 2H, SCH$_2$), 4.80 (dq, 1H, *J* = 17.2, 1.5 Hz, CH$_2$CH=CH$_2$), 5.08 (dq, 1H, *J* = 10.4, 1.5 Hz, CH$_2$CH=CH$_2$), 5.72 (ddt, 1H, *J* = 17.2, 10.4, 5.1 Hz, CH$_2$CH=CH$_2$), 6.52 (d, 1H, *J* =3.4 Hz, H-fur), 6.97–7.03 (m, 2H, $C_6H_4OC_5H_{11}$), 7.05 (d, 1H, *J* =3.4 Hz, H-fur), 7.53 (ddd, 1H, *J* =8.3, 7.0, 1.1 Hz, C_6H_4), 7.73–7.79 (m, 2H, C_6H_4), 8.08 (s, 1H, =CH, pyr.), 8.11 (dd, 1H, *J* = 8.6, 1.2 Hz, C_6H_4), 8.19–8.24 (m, 2H, $C_6H_4OC_5H_{11}$), 12.66 (br, 1H, COOH). ^{13}C NMR, δ(ppm): 13.6 (CH_3), 21.9 (CH_2), 27.7 (CH_2), 28.4 (CH_2), 29.9 (SCH_2), 46.3 (NCH_2), 67.3 (OCH_2), 110.6 (=CH_2), 114.1 (2 CH), 117.5 (CH), 117.8 (CH), 118.8 (CH), 124.3, 124.8 (CH),126.3 (CH), 128.4 (2 CH), 129.4 (CH), 129.5 (CH), 130.2, 131.4 (CH), 133.1, 144.8, 148.1, 149.7, 152.0, 153.6, 155.0, 158,7, 160. HRMS [ESI$^+$, MeOH]: calcd for $C_{31}H_{31}O_4N_4S$ $[M+H]^+$ 555.2061, found 555.2057. El. anal. calcd. for $C_{31}H_{30}N_4O_4S$: C 67.13; H 5.45; N 10.10; S 5.78; Found: C 66.95; H 5.37; N 10.18; S 5.66.

Video S1. Dynamic view of the superposition of small compounds bound to EGFR.

Video is accessible at the site: https://www.dropbox.com/s/1n3djdy7a2iy6uq/movie.gif?dl=0

Compounds VM23 (orange), VM25 (pink), and VM26 (green) and gefitinib (yellow) are shown as thick string and amino acids Arg803, Arg841, Cys797, and Met766 are shown as thin string in colors corresponding to the interacting compounds.

© 2019 by the authors. Licensee MDPI, Basel, Switzerland. This article is an open access article distributed under the terms and conditions of the Creative Commons Attribution (CC BY) license (http://creativecommons.org/licenses/by/4.0/).
